# A Reactive Antagonist Strategy: Cysteine‐Directed Covalent Molecular Glue in Plant Hormone Receptor Regulation

**DOI:** 10.1002/advs.202517463

**Published:** 2026-01-08

**Authors:** Minoru Ueda, Kotaro Matsumoto, Takao Nomura, Yousuke Takaoka, Taichi Okumura, Wataru Kozaki, Hikaru Hoshikawa, Yuho Nishizato, Andrea Chini, Roberto Solano, Katsumi Maenaka

**Affiliations:** ^1^ Department of Chemistry, Graduate School of Science Tohoku University Sendai Japan; ^2^ Department of Molecular and Chemical Life Sciences, Graduate School of Life Sciences Tohoku University Sendai Japan; ^3^ Center For Research and Education on Drug Discovery, Faculty of Pharmaceutical Sciences Hokkaido University Sapporo Japan; ^4^ Plant Molecular Genetics Department, National Centre for Biotechnology (CNB) Consejo Superior de Investigaciones Cientificas (CSIC) Madrid Spain

**Keywords:** COI1‐JAZ co‐receptor, JA‐Ile, molecular glue, plant hormone, PPI

## Abstract

Controlling protein–protein interactions (PPIs) remains a challenge in modern chemistry. In plants, jasmonoyl‐L‐isoleucine (JA‐Ile) acts as a molecular glue that facilitates PPIs between the F‐box protein COI1 and JAZ repressors, leading to transcriptional reprogramming. However, the functional redundancy of JAZ proteins complicates biological studies. To overcome this, we introduce a reactive antagonist (RA) strategy that selectively induces COI1‐JAZ interactions for a Cys‐mutated JAZ mutant while inhibiting interactions with wild‐type JAZs. We designed and synthesized COR‐oxime derivatives, identifying *O*‐bromo‐ethyl COR‐oxime as an optimal RA, functioning as a Cys‐directed covalent molecular glue. Mass spectrometry and fluorescence anisotropy assays confirmed its selective covalent binding to Cys‐mutated JAZ1, leading to targeted degradation in *Arabidopsis*. This strategy enables precise functional dissection of JAZ subtypes without affecting endogenous pathways. Given the genetic redundancy in many biological systems, the RA strategy provides a powerful chemical biology tool for dissecting complex signaling networks beyond plant hormone research.

## Graphical abstract

The plant hormone jasmonate is perceived by COI1‐JAZ co‐receptors comprising a single F‐box protein COI1 and multiple JAZ repressors with overlapping functions, complicating genetic analysis. We present a reactive antagonist (RA) strategy that selectively promotes COI1 binding to a cysteine‐engineered JAZ variant while blocking wild‐type JAZs, enabling subtype‐specific activation of jasmonate signaling.

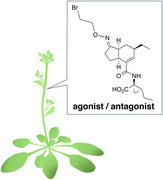

## 1 Introduction

Controlling protein–protein interactions (PPIs) using molecular technologies remains a significant challenge in modern chemistry [1, 2]. Recently, synthetic PPI inducers, such as proteolysis‐targeting chimeras (PROTACs) and molecular glues, have garnered considerable attention [3, 4, 5, 6]. Interestingly, most physiological functions in plants are regulated by phytohormones [7, 8, 9], which are among the oldest known endogenous molecular glues [10]. One such plant hormone, (3*R*, 7*S*)‐jasmonoyl‐L‐isoleucine (JA‐Ile, **1**), induces plant defense responses against pathogenic bacteria and herbivorous insects [11, 12, 13, 14]. Within plant cells, JA‐Ile functions as a molecular glue, facilitating PPI between the F‐box protein CORONATINE INSENSITIVE 1 (COI1) and JASMONATE‐ZIM DOMAIN (JAZ) repressor proteins [15, 16]. The subsequent degradation of JAZ via the 26S‐proteasome pathway derepresses transcription factors (TFs), leading to the expression of jasmonate‐responsive genes and transcriptional reprogramming of plant cells.

The COI1‐JAZ co‐receptor system in *Arabidopsis thaliana* consists of 13 JAZ proteins and a single COI1 protein, with each JAZ subtype regulating unique and overlapping biological functions. This functional redundancy often compensates for the loss of a single *JAZ* gene in a single *JAZ* knockout mutant, complicating the genetic study for the functional dissection of individual *JAZ* in the complex JA‐Ile‐induced transcriptional network [17, 18].

Chemical genetics provides a promising approach to address the issues arising from genetic redundancy in jasmonate signaling. We previously developed NOPh, which was designed from the JA‐Ile mimic coronatine (COR, **2**), as the first subtype‐selective agonist of the COI1‐JAZ co‐receptor [19], but its selectivity was insufficient for fully dissecting the complexity of jasmonate signaling.

**FIGURE 1:**
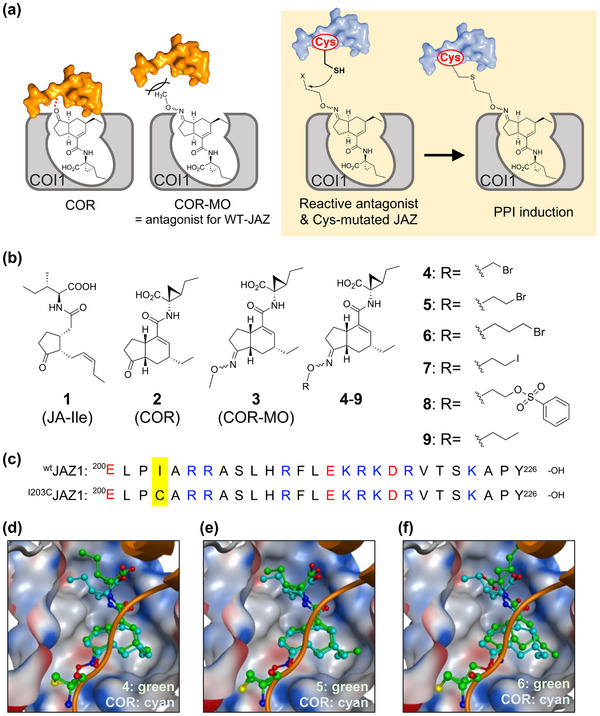
Design strategy of the reactive antagonist for specific PPI induction of the cysteine‐mutated JAZ and COI1. (a) Schematic of the design of the reactive antagonist and cysteine‐mutated JAZ based on COR‐MO and wtJAZ1: COI1‐COR‐MO complex, COR‐MO as an antagonist, and RA functioning as a Cys‐directed covalent molecular glue. (b) Chemical structures of naturally occurring ligands (JA‐Ile (**1**), COR (**2**)), the reported COI1‐JAZ antagonist COR‐MO (**3**), and the designed RA (**4**–**9**). (c) Amino acid sequence of the Jas motif of JAZ1 (^wt^JAZ1) and cysteine‐mutated JAZ1 (^I203C^JAZ1). (d–f) Superimposed structures of covalent docking analysis of the reactive antagonist (green) and the original ligand **2** (cyan); (d) **4**, (e) **5**, (f) **6**, respectively. The RMSD values for these ligands compared to **2** were 1.63 Å (**4**, **d**), 1.07 Å (**5**, **e**), and 2.34 Å (**6**, **f**), respectively.

Here, we introduce a reactive antagonist (RA) strategy that enables the selective formation of a co‐receptor pair between COI1 and a specific JAZ subtype. This approach allows for the precise activation of JAZ subtype‐specific signaling, overcoming the severe genetic redundancy of JAZ genes, which previously hindered such studies (Figure 1a).

## 2 Results and Discussion

COR‐MO (**3**, Figure 1b), a potent antagonist of the COI1‐JAZ co‐receptor, was previously designed and synthesized based on the crystal structure of the COI1‐COR‐JAZ1 complex (Figure 1a) [20]. Compound **3** binds COI1, but its methyl oxime moiety protrudes from the COI1‐**3** complex, obstructing interaction with JAZ proteins [21, 22]. We hypothesized that incorporating an electrophilic reactive functionality (e.g., a halogen atom) into the methyl oxime moiety of **3** (Reactive Antagonist, RA, Figure 1a) would enable covalent bond formation with a modified JAZ protein, in which a nucleophilic Cys residue replaces an existing amino acid at an appropriate sequence position. This interaction is feasible because almost all JAZ subtypes lack a Cys residue in the Jas motif (Figure S1c,d), which is critical for COR‐mediated PPI between COI1 and JAZ.

Optimizing the RA strategy requires careful molecular design to ensure an effective combination of RA and Cys‐modified JAZ. We modified **3** to generate an optimized RA that (i) functions as a potent antagonist for wild‐type (WT) JAZs and (ii) possesses a reactive functionality positioned for covalent bond formation with Cys‐containing JAZ. We identified Ile203 of JAZ1 as the most suitable site for Cys substitution because in silico docking models predicted its proximity to the oxime moiety of **3** (Figure 1c, Figure S1a,b). Furthermore, the mutation in Ile203 is predicted not to affect the function of JAZ because it is not well conserved among the 98 JAZ subtypes across 10 plant species (Figure S1c,d).

Based on these insights, we designed *O*‐bromo‐methyl (**4**), *O*‐bromo‐ethyl (**5**), and *O*‐bromo‐propyl (**6**) COR‐oximes as candidate RAs (Figure 1b). We did not employ acrylamide‐based warheads because, despite their small size, they often display broad off‐target reactivity. [23] Instead, we selected a moderately reactive bromoethyl warhead to ensure more reliable and selective Cys‐modification. We utilized in silico covalent docking simulations [24] to evaluate their binding potential. The Root Mean Square Deviation (RMSD) values, compared to the original COR (RMSD = 0.973 Å), indicated that **5** in combination with ^I203C^JAZ1 (RMSD = 1.07 Å) would form the most stable complex post‐covalent bond formation (Figure 1d–f). Based on these results, we synthesized **5** and its derivatives **7** and **8** (iodo‐ and benzenesulfonate‐conjugated oximes) to optimize reactivity, as well as non‐reactive COR‐*O*‐propyl oxime (**9**) as a negative control.

To establish a proof‐of‐principle, we examined the potency of compounds **5**, **7**, and **8** as reactive antagonists (RAs) in a model system comprising COI1 and fluorescein‐conjugated JAZ1 degron peptide (Fl‐JAZ1P). Our previous studies confirmed that Fl‐JAZ1P binds to COI1 with comparable affinity to the full‐length JAZ1 protein in the presence of coronatine (COR) [19]. Fluorescence anisotropy (FA) assays further demonstrated that Cys‐mutated Fl‐JAZ1P (Fl‐^I203C^JAZ1P) facilitated COR‐mediated protein‐protein interaction (PPI) with COI1 as effectively as Fl‐^Wt^JAZ1P (Figure S2, *K*_d_ values of 6.6 and 4.2 nM, respectively). Therefore, subsequent experiments are independent of affinity differences between Fl‐^Wt^JAZ1P and Fl‐^I203C^JAZ1P in COI1‐JAZ complex formation, primarily reflecting variations in reactivity among COR oximes.

**FIGURE 2:**
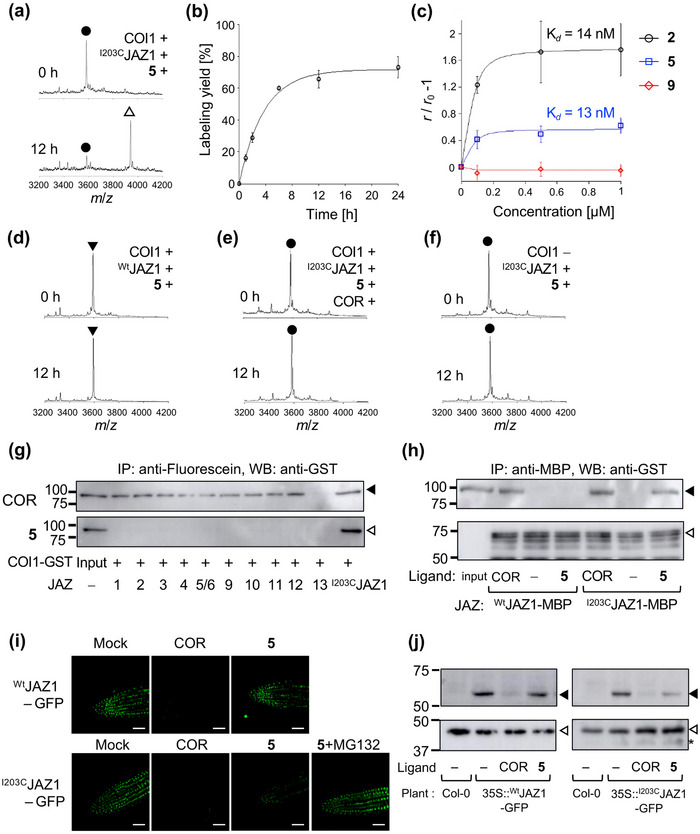
Proof‐of‐principle experiments of the reactive antagonist strategy. (a) MALDI‐TOF MS analyses of the in vitro labeling of Fl‐^I203C^JAZ1P (1 µM) with GST‐COI1 (1 µM) and **5** (1 µM). The reaction mixtures were incubated for 0 h (top) or 12 h (bottom) at 22°C. (b) The time dependency of the labeling yields of Fl‐^I203C^JAZ1P (1 µM) with GST‐COI1 (1 µM) and **5** (1 µM), monitored by MALDI‐TOF MS. Results shown are mean with SD. (c) Dose dependency of fluorescence anisotropy (FA) changes of Fl‐^I203C^JAZ1P (1 µM) with GST‐COI1 (1 µM) and each ligand (**2** (black circle), **5** (blue square), or **9** (red diamond) (0–1 µM), respectively). The reaction mixture was incubated for 12 h at 22°C, then the solution was diluted 10 times, and the FA values were detected. Results shown are mean with SD. (d–f) Control experiments of the in vitro labeling of (a); (d) ^wt^JAZ1 instead of ^I203C^JAZ1, (e) in the presence of **2** (50 µM), (f) in the absence of GST‐COI1. The reaction mixtures were incubated for 0 h (top) or 12 h (bottom) at 22°C. (g) Pull‐down assay of GST‐COI1 (5 nM) with each Fl‐JAZP (10 nM) in the presence of **2** (COR, 100 nM, top) or **5** (100 nM, bottom). Anti‐Fl antibody was used for immunoprecipitation, and anti‐GST‐HRP was used for chemiluminescence detection of GST‐COI1. The experiments were repeated three times with similar results. (h) Pull‐down assay of GST‐COI1 (5 nM) with full‐length JAZ1‐MBP (40 nM, ^wt^JAZ1‐MBP or ^I203C^JAZ1‐MBP) in the absence of presence of **2** (COR, 100 nM) or **5** (100 nM). Amylose resin was used for pull‐down. Anti‐GST‐HRP was used for chemiluminescence detection of GST‐COI1 (black triangles). Anti‐MBP antibodies and rat‐IgG‐HRP were used to visualize JAZ1‐MBP protein levels as the input materials (white triangles). The experiments were repeated three times with similar results. (i) Fluorescence live imaging of 35S::JAZ1‐GFP treated with mock, **2** (COR, 1 µM) or **5** (1 µM) in the absence or presence of MG132 (100 µM) for 2 h at 22°C; top: 35S::^wt^JAZ1‐GFP, bottom 35S::^I203C^JAZ1‐GFP. The experiments were repeated three times with similar results (shown in Figure S6c). Scale bar 50 µm (j) Western blotting analyses of 35S::JAZ1‐GFP treated with mock, **2** (COR, 1 µM) or **5** (1 µM) for 2 h at 22°C. Anti‐GFP‐HRP (top, 56 kDa, black triangles) or anti‐b‐actin (bottom, 40 kDa, white triangles) was used for chemiluminescence detection. The experiments were repeated three times with similar results.

To evaluate the reactivity of **5**, **7**, and **8**, we performed mass spectrometry (MS) analysis. Labeling reactions using **5** in the presence of COI1 yielded an ion peak at *m*/*z* 3946, corresponding to the **5**‐labeled Fl‐^I203C^JAZ1P. This peak appeared after 30 min of incubation, with the reaction yield reaching 65% after 12 h (Figure 2a,b, Figure S3a). Under the same conditions, FA increased markedly following labeling, similar to COI1‐COR‐Fl‐^Wt^JAZ1P (Figure 2c). The *K*_d_ values were calculated as *K*_d_ = 13 nM for **5** and *K*_d_ = 14 nM for **2** (COR) (Figure 2c). MS/MS analysis confirmed the labeling site at ^203^Cys (Figure S3b). Additionally, the labeling yield of **7** was comparable to that of **5**, whereas **8** exhibited significantly lower labeling efficiency (Figure S4a–d). Considering in vivo stability, **5** was selected as the RA for subsequent experiments. No labeling or FA changes were observed with non‐reactive compound **9** under identical conditions, validating the effectiveness of our RA strategy (Figure 2c, Figure S4c,d). Furthermore, when Fl‐^Wt^JAZ1P (lacking Cys) was used instead of Fl‐^I203C^JAZ1P, no RA‐labeled peak was detected (Figure 2d). Similarly, no reaction occurred between Fl‐^I203C^JAZ1P and **5** in the presence of excess COR or absence of COI1 (Figure 2e,f).

To further investigate specificity, we tested the noncanonical ^WT^JAZ13, which contains Cys at the same position as Fl‐^I203C^JAZ1P. No labeling by **5** was observed for Fl‐^wt^JAZ13P, consistent with previous findings that JAZ13 does not form a COI1‐JAZ13‐COR complex due to the absence of a canonical Jas sequence which is necessary for COI1‐JAZ formation (Figure S5) [25]. Additionally, pull‐down assays after pre‐incubation of COI1 and **5** with Fl‐JAZ1P supported the FA results, revealing interaction exclusively for ^I203C^JAZ1P, with no interaction observed for functional ^Wt^JAZPs (JAZ1‐6/9‐12) or ^wt^JAZ13P (Figure 2g, Figure S6a). JAZ7/8 were excluded from the analysis due to their non‐canonical Jas motifs and minimal affinity for COI1 in the presence of JA‐Ile/COR [26, 27, 28]. These results confirm that **5** functions as a specific agonist for the COI1‐^I203^CFl‐JAZ1P pair. Moreover, pull‐down assays using full‐length JAZ proteins demonstrated that **5** induces PPI between COI1 and full‐length ^I203C^JAZ1 (Figure 2h, Figure S6b).

Finally, the *in planta* effects of **5** were examined using *Arabidopsis* transgenic reporter lines expressing *35S::^wt^JAZ1‐GFP* and *35S::^I203C^JAZ1‐GFP*. Treatment with **5** resulted in significant degradation of ^I203C^JAZ1‐GFP, similar to COR, whereas no degradation was observed in the *35S::^wt^JAZ1‐GFP* line (Figure 2i,j, Figure S6d). The degradation was suppressed by MG132, a proteasome inhibitor (Figure 2i, Figure S6c). These findings confirm that **5** facilitates PPI between COI1 and full‐length ^I203C^JAZ1, triggering in vivo degradation of ^I203C^JAZ1‐GFP in *Arabidopsis*, while ^wt^JAZ1‐GFP remains unaffected (Figure 2i, Figure S6c).

**FIGURE 3:**
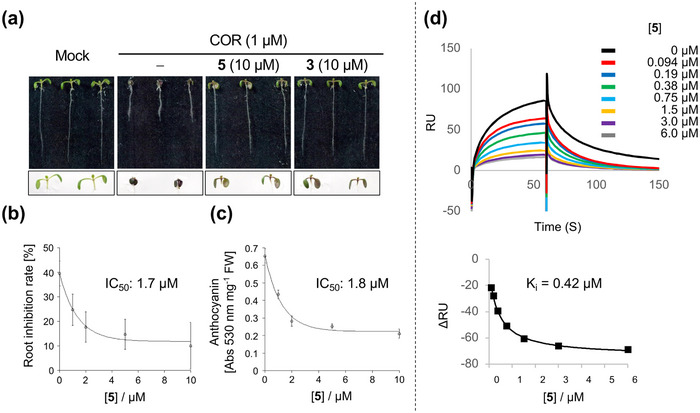
Antagonist activity of RA. (a) *Arabidopsis* WT seedlings grown for 6 days on 1/2 MS medium containing mock condition or COR (**2**, 1 µM) in the absence or presence of **5** (10 µM) and COR‐MO (**3**, 10 µM). The representative images of the aerial part are shown at the bottom. (b, c) Root length inhibition rate (b) and quantification of anthocyanin accumulation (c) of 6‐day‐old seedlings grown on 1/2 MS medium containing COR in the absence or presence of **5** (0–10 µM). The results shown are the mean with SD. The experiments were repeated three times with similar results. Abs, absorbance, FW, fresh weight. (d) SPR assay of **5** with COI1 (0.24 µM) and JA‐Ile (10 µM) using MBP‐JAZ1‐His_6_ chip. The response units (RU) decreased in a dose dependent manner of **5**. Sensorgrams are in top panel and ΔRU dose‐response curves of **5** are in bottom one. The association and dissociation times were set as 60 and 180 s, respectively.

Next, we evaluated the potency of **5** as an antagonist against JA‐Ile perception by COI1‐JAZ co‐receptors. Two hallmark JA‐Ile‐induced responses, anthocyanin accumulation and root growth inhibition, were strongly suppressed by **5**, similar to compound **3** (Figure 3a–c, Figure S7). To confirm that this antagonistic effect results from interference with JA‐Ile‐induced COI1‐JAZ co‐receptor formation, we performed pull‐down and FA assays using functional JAZs (JAZ1‐6/9‐12). The SPR assay using COI1 and MBP‐JAZ1‐His_6_ proteins revealed a strong dose‐dependent antagonistic effect of **5** (Figure 3d, Figure S8) [29, 30]. Derived from SPR data, the *K*_i_ value of **5** was estimated to be 0.42 mM based on the decline in response units upon COI1/**5** complex formation, fitted to a 1:1 Langmuir binding model. FA assays further confirmed that **5**, like COR‐MO, functions as a potent antagonist for COI1‐JAZ1‐6/9‐12 pairs, all of the functional COI1‐JAZ co‐receptors in *A. thaliana* (Figures S9, S10). These results demonstrate that **5** effectively inhibits COI1‐JAZ interactions across all functional COI1‐JAZ pairs. Thus, our findings establish that **5** functions as an RA that induces the selective degradation of ^I203C^JAZ1 without affecting ^WT^JAZ proteins in ^I203C^JAZ1‐expressing *A. thaliana*.

**FIGURE 4:**
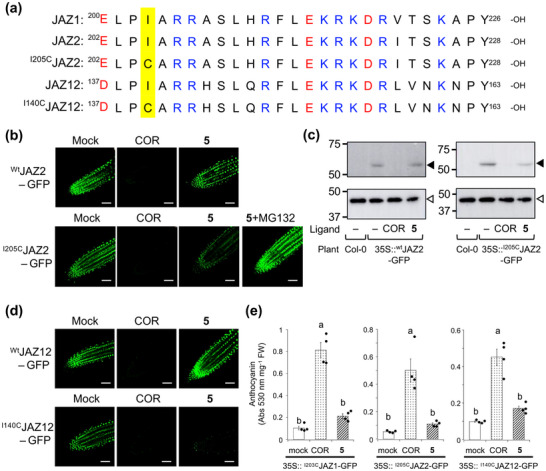
RA strategy can apply to specific PPI induction of COI1 and any JAZ subtype. (a) The amino acid sequence of the Jas motif of JAZ1, 2, 9, 12 and cysteine‐mutated JAZ (^I205C^JAZ2, ^Q221C^JAZ9, and ^I140C^JAZ12). (b) Fluorescence live imaging of 35S::JAZ2‐GFP treated with mock, **2** (COR, 1 µM) or **5** (1 µM) in the absence or presence of MG132 (100 µM) for 2 h at 22°C; top: 35S::^wt^JAZ2‐GFP, bottom 35S::^I205C^JAZ2‐GFP. The experiments shown in this figure were repeated three times with similar results. Scale bar 50 µm (c) Western blotting analyses of 35S::JAZ2‐GFP treated with mock, **2** (COR, 1 µM) or **5** (1 µM) for 2 h at 22°C. Anti‐GFP‐HRP (top, 56 kDa, black triangles) or anti‐b‐actin (bottom, 40 kDa, white triangles) was used for chemiluminescence detection. (d) Fluorescence live imaging of 35S::JAZ12‐GFP treated with mock, **2** (COR, 1 µM) or **5** (1 µM) for 2 h at 22°C. The experiments shown in this figure were repeated three times with similar results. Scale bar 50 µm. (e) Quantification of anthocyanin accumulation of 6‐day‐old seedlings grown on 1/2 MS medium in the absence (mock) and presence of COR (**2**, 1 µM) or **5** (1 µM) (n = 4). The results shown are the mean with SD. The significant differences were evaluated by one‐way ANOVA/Tukey HSD post hoc test. Different letters represent a significant difference at *p* < 0.05. The experiments were repeated three times with similar results. Abs, absorbance, FW, fresh weight.

We next investigated the potential of **5** as a specific agonist for other Cys‐mutated JAZ subtypes. In vitro analyses were performed on COI1‐^I205C^JAZ2, which corresponds to ^I203C^JAZ1 (Figure 4a). MS and pull‐down analyses confirmed that **5** bound to and reacted with Fl‐^I205C^JAZ2P (Figure S11). Moreover, **5** specifically induced PPI between COI1 and full‐length ^I205C^JAZ2, whereas no PPI was observed between COI1 and full‐length ^Wt^JAZ2 (Figure S12).

To assess *in planta* selectivity, fluorescent imaging and western blotting analyses were conducted on *Arabidopsis* transgenic lines overexpressing Cys‐mutated JAZ‐GFP [16], I205CJAZ2‐GFP‐OE (*35S::^I205C^JAZ2‐GFP*), and ^wt^JAZ2‐GFP‐OE (*35S::^wt^JAZ2‐GFP*) as a control. COR induced JAZ‐GFP degradation in ^wt^JAZ2‐GFP‐OE, whereas both COR and **5** triggered degradation in ^I205C^JAZ2‐GFP‐OE (Figure 4b,c, Figure S13). Degradation of ^I205C^JAZ2‐GFP was suppressed by MG132 (Figure 4b). Additionally, we verified **5**‐triggered degradation of Cys‐mutated JAZ12, belonging to a different subclade from JAZ1/2 in the phylogenetic analysis (Figure S14a). Treatment with **5** or COR resulted in significant degradation of ^I140C^JAZ12‐GFP in the ^I140C^JAZ12‐GFP‐OE transgenic line, whereas ^wt^JAZ12‐GFP remained unaffected (Figure 4d, Figure S14b,c). These findings suggest that **5** functions as an RA specifically targeting any Cys‐mutated JAZ subtype in *Arabidopsis*.

Furthermore, **5** did not induce typical JA responses, such as anthocyanin accumulation or growth inhibition, which are strongly triggered by COR‐mediated degradation of ^wt^JAZs (Figure 4e, Figure S15). These results confirm that **5** does not impact ^wt^JAZ subtypes in Cys‐mutated JAZ1/2/12‐GFP‐OE lines, highlighting its selectivity for Cys‐mutated JAZ proteins.

## 3 Conclusion

In plant science, novel and specific antagonists/agonists for plant hormone receptors have been developed as valuable chemical tools [31]. For example, a bump‐and‐hole approach successfully enabled the subtype‐selective activation of the TIR1/ARF‐AUX/IAA auxin co‐receptor system [32]. This strategy involved using a modified synthetic auxin and a genetically engineered TIR1 receptor, thereby activating TIR1/ARF‐derived signaling without interfering with endogenous auxin or TIR1/AFBs. However, the functional redundancy of AUX/IAA repressors remained unresolved, hindering complete dissection of the signaling network.

**FIGURE 5:**
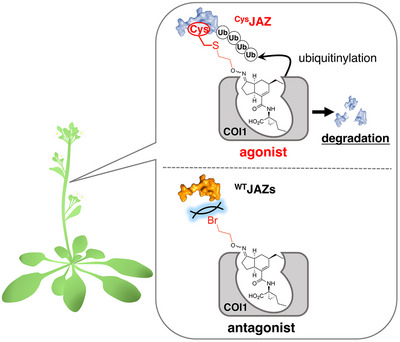
Whole picture of Reactive Antagonist strategy.

We have developed a unique reactive antagonist (RA) strategy that specifically induces COI1‐JAZ co‐receptor formation for Cys‐mutated JAZ while inhibiting COI1‐JAZ co‐receptor formation for co‐existing wild‐type JAZ (^WT^JAZ) (Figure 5). JAZ proteins are highly genetically redundant transcriptional repressors, and until now, the only way to elucidate the function of specific JAZ subtypes has been through the generation of multiple knockout mutants targeting many of the 13 JAZ isoforms. Our strategy, in principle, enables selective functional interrogation of any desired JAZ subtype among the 13 isoforms.

We previously reported the development of JAZ subtype‐selective agonists using a chemical library of **2**‐stereoisomers [33]. However, we found that this approach alone does not readily allow rational design of agonists that selectively act on any desired JAZ subtype among the 13 family members. In contrast, in the RA strategy presented in this study, formation of the COI1–JAZ co‐receptor complex is restricted to JAZ subtypes in which a Cys‐residue has been pre‐introduced. Thus, a single JAZ isoform can be chosen from the 13 members as the target, and co‐receptor assembly and subsequent degradation can be induced selectively for that subtype. Moreover, the RA behaves as an antagonist toward wild‐type JAZ proteins that lack the engineered cysteine, thereby inhibiting endogenous JA‐Ile–dependent COI1–JAZ complex formation and downstream signaling. As a result, signaling derived from only the selected JAZ isoform can be selectively activated, functionally insulated from endogenous JA‐Ile signaling, which represents a major advantage of this strategy.

Many genes with essential biological functions exhibit genetic redundancy [34]. Since this approach is applicable to other cases where antagonists targeting genetically redundant proteins are available, it holds broad potential as a general strategy. Therefore, the reactive antagonist strategy represents a powerful chemical biology approach that complements conventional genetic methodologies in studying genetically redundant biological functions.

## 4 Experimental Section/Methods

### 4.1 In Silico Docking Study

**SCHEME 1:**
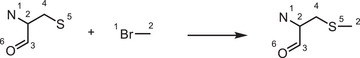
The reaction between alkyl bromide and Cysteine for MDL RD file in MOE.

In silico covalent docking analyses were carried out using MOE 2020.0901 software (Chemical Computing Group, Montreal, CA). Initially, the 3D structural homology modeling of COI1‐**5**‐^I203C^JAZ1 was prepared from original structure of COI1‐COR‐JAZ1 (PDB ID: 3OGM) with I203C substitution. After addition of polar hydrogens, the whole structure was calculated with energy minimization with Amber10 EHT force field. The geometrical parameters for 3D structures of the compounds were optimized, and partial charges were calculated before docking. The MDL RD file (rdf file) defining the reaction between alkyl bromide and thiol of cysteine was prepared as shown in Scheme 1. The covalent docking simulation between the three alkyl bromide compounds and COI1‐^I203C^JAZ1 was carried out under the following conditions; forcefield: Amber10 EHT, Reactive site: 203Cys residue, Reaction: the above‐mentioned original rdf file, Refinement: Induced fit, GBV/WSA dG. RMSD was calculated with the original SVL program.

### 4.2 MALDI‐TOF MS Experiments

Purified COI1‐GST, ASK1, and Fl‐JAZ peptides were prepared by using previous method.[19, 35] Purified COI1‐GST (1 µM) with ASK1, Fl‐JAZ peptides (1 µM), **5** (1–10 µM), and IP5 (1 µM) were dissolved in 22 µL Buffer A (50 mM Tris‐HCl, pH 7.8, 100 mM NaCl, 10 % glycerol, 0.1 % Tween‐20, 1 mM TCEP (tris(2‐carboxyethyl)phosphine), and incubated at r.t. for 24 h. 3.5 µL of the samples were corrected and purified by ZipTip (C18, Merck KGaA, Germany) at each time point (0, 1, 2, 6, 12, 24 h) and were characterized by MALDI‐TOF MS. Labeling yield (Y) was calculated by the following equation: Y = B/(A+B), where A and B are the baseline corrected intensities of intact JAZ peptide and labeling JAZ peptide, respectively. (*n* = 3, values represent mean ± SD.)

### 4.3 LC‐MS/MS Analysis for Identification of Labeling Site

Purified COI1‐GST (1 µM) with ASK1, Fl‐^I203C^JAZ1 peptide (1 µM), **5** (1 µM), and IP5 (1 µM) were dissolved in 270 µL Buffer B (50 mM Tris‐HCl, pH 7.8, 100 mM NaCl, 10 % glycerol, 0.1 % Tween‐20, 1 mM TCEP (tris(2‐carboxyethyl)phosphine), 1 mM PMSF, 1 mM EDTA), and incubated at r.t. for 23 h. The labeling reaction was monitored by MALDI‐TOF MS analysis. Then, **5** (1 µM) was again added and further incubated at r.t. for 20 h for completion of the reaction. Further experiments were conducted by Intégrale Co., Ltd. (Japan). The labeled ^I203C^JAZ1 peptide was precipitated by trichloroacetic acid (TCA) and the precipitation was redissolved with Buffer C (20 mM Tris‐HCl, pH 8.0). Trypsin was added to the peptide solution and incubated at 37°C for 20 h, then desalted and the digested peptide solution was analyzed using a Q Exactive plus Mass Spectrometer (Thermo Fisher Scientific Inc., USA) equipped with an EASY‐nLC 1200 LC system (Thermo Fisher Scientific Inc., USA). The peptide solution was injected onto a 75 µm × 150 mm EASY‐spray column (Thermo Fisher Scientific Inc., USA). Solvent A (0.1% formic acid in water) and solvent B (0.1% formic acid in 80% aqueous acetonitrile solution) were prepared for the analysis. Peptide was separated using gradients of 5%–40% solvent B in 10 min, 40%–100% solvent B in 2 min, and 100% solvent B in 8 min and electrosprayed into the Q Exactive plus Mass Spectrometer at a flow rate of 300 nL min^−1^. The collision energy for performing MS/MS was set to 27%. The exclusion list was generated on the basis of MASCOT searching results and set threshold value to 50% to identify target peptide.

### 4.4 Pull‐Down Experiments Using Fl‐JAZ Peptides

For the agonist activity experiment, purified COI1‐GST (500 nM), Fl‐JAZ peptides (1 µM), **5** (1 µM), and IP5 (1 µM) were dissolved in 5 µL pull‐down buffer (50 mM Tris‐HCl, pH 7.8, 100 mM NaCl, 10 % glycerol, 0.1 % Tween‐20, 1 mM TCEP), incubated at r.t. for 12 h, and added 495 µL cold pull‐down buffer. For the antagonist activity experiment, purified COI1‐GST (5 nM), Fl‐JAZ peptides (10 nM), and, 7‐*iso*‐JA‐Ile (100 nM), **5** (0.1–30 µM), and IP5 (100 nM) were dissolved in 500 µL pull‐down buffer and incubated on ice for 1 h. After incubation in both experiments, the samples were combined with anti‐fluorescein antibody (GeneTex, GTX26644, 0.2 µL) and incubated for 8–16 h at 4°C with rotation. After incubation, the samples were combined with Protein G Mag Sepharose Xtra (GE Healthcare, 15 µL in pull‐down buffer slurry). After 2–4 h incubation at 4°C with rotation, the samples were washed 3 times (for the agonist experiments) or 10 times (for the antagonist experiments) with 500 µL cold wash buffer (0.1% Tween‐20 in PBS buffer). The washed beads were resuspended in 50 µL SDS‐PAGE loading buffer containing 100 mM dithiothreitol (DTT). After heating for 10 min at 60°C, the samples were subjected to SDS‐PAGE and analyzed by western blotting with iBind Western System (Thermo Fisher Scientific K.K., USA). The bound COI1‐GST protein was detected using anti‐GST‐HRP conjugate (#RPN1236, GE Healthcare, 5,000‐fold dilution in western buffer (10% (v/v) Blocking One (Nacalai tesque) in wash buffer)).

### 4.5 Pull‐Down Experiments Using Full‐Length JAZ Proteins

The plasmids for expression of cys‐mutated MBP‐fused JAZ1/2 proteins were generated from *pMal‐C2‐JAZ1/2* ^S4^ by conventional inverse PCR method with the primers 11 and 12 for JAZ1 or primers 5 and 6 for JAZ2 (Table S1). All the MBP‐fused JAZ proteins (JAZ1, ^I203C^JAZ1, JAZ2, ^I205C^JAZ2) were expressed in *Escherichia coli* cells (BL21(DE3)) and purified by amylose resin (New England Biolabs) as described previously.[19, 36] In each pull‐down experiment, purified COI1‐GST (5 nM) with ASK1, COR (100 nM) or **5** (100 nM), and IP5 (100 nM) are dissolved in 500 µL pull‐down buffer (50 mM Tris‐HCl, pH 7.8, 100 mM NaCl, 10 % glycerol, 0.1 % Tween‐20, 20 mM 2‐mercaptoethanol) and added to amylose resin‐bound MBP‐JAZ (20 µL suspension of amylose resin containing 40 nM of MBP‐JAZs). After 1‐2 h incubation at 4°C under rotation, the samples were washed three times with 500 µL cold pull‐down buffer. The washed beads were resuspended in 50 µL SDS‐PAGE loading buffer containing 20 mM maltose. After boiling for 10 min at 60°C, the samples were loaded on SDS‐PAGE and analyzed by western blotting. The bound COI1‐GST protein was detected using anti‐GST‐HRP conjugate (#RPN1236, GE Healthcare, 5,000‐fold dilution in western buffer (10% (v/v) Blocking One in PBS containing 0.1% Tween‐20)). MBP‐JAZ was detected using rat anti‐MBP anti body (016‐24141, Wako, 5000‐fold dilution in western buffer) and goat anti‐rat‐IgG‐HRP antibody (#sc‐2032, Santa Cruz Biotechnology, 20 000‐fold dilution in western buffer).

### 4.6 GFP Imaging for JAZ‐GFP Overexpressing Arabidopsis Seedling

For GFP imaging experiments, 4‐d‐old seedlings of *P_35S_::JAZ‐GFP* or *P_35S_::cysJAZ‐GFP* were transferred in 1/2 MS (0.5% sucrose) liquid medium containing compounds (COR (**2**) or RA (**5**) 1 µM) for 2 h. After compound treatments, nuclear localized GFP fluorescence was observed by LSM700 laser scanning confocal microscope (Carl Zeiss Co., Ltd., GE) with using laser line 488 nm (intensity: 2%), with 20x/0.8 M27 objective lens, variable secondary dichroic beam splitter (setting 493 nm), no emission filter, and pinhole: 70 µm.

For western blotting analyses, 6 seedlings of 4‐d‐old *P_35S_::JAZ‐GFP or P_35S_::cysJAZ‐GFP* were transferred in 1/2 MS (0.5% sucrose) liquid medium containing compounds (COR, RA 1 µM) for 2 h. Then seedlings were grounded on liquid nitrogen, and homogenized them on ice with 50 µL extraction buffer (50 mM Tris‐HCl, pH 7.4, 100 mM NaCl, 10% (v/v) glycerol, 0.1% (v/v) Tween‐20, 1 mM DTT, 1 mM phenylmethylsulfonylfluoride (PMSF), complete protease inhibitor (Roche) and 50 µM MG132). Centrifuged at 4°C, 15 000 g for 10 min and collected supernatant. 2 µL extract were used for Bradford assay to calculate the protein concentration. 30 µL extract were mixed with 10 µL 4 × SDS‐PAGE loading buffer. After heating at 60°C for 10 min, the samples were subjected to SDS‐PAGE and analyzed by western blotting. JAZ‐GFP protein was detected using anti‐GFP‐HRP conjugate (#130‐091‐833, Miltenyi Biotec Co., North Rhine‐Westphalia, DE) 5000‐fold dilution in PBS buffer containing 0.1% Tween‐20). b‐actin was detected using anti‐b‐actin‐mouse‐IgM (#60008‐1‐Ig, Proteintech Group, Illinois, US), 1000‐fold dilution in western buffer (10% (v/v) Blocking One (Nacalai tesque) in PBS buffer containing 0.1% Tween‐20)) and anti‐mouse IgM‐HRP (G0417, Tokyo Chemical Industry Co., Ltd., Tokyo, Japan) 20 000‐fold dilution in western buffer (10% (v/v) Blocking One (Nacalai tesque) in PBS buffer containing 0.1% Tween‐20)) for a control the amount of protein.

### 4.7 Root Length Measurements

2‐day‐old seedlings of Col‐0 were transferred on 1/2 MS plate in the presence or absence of 1–10 µM of each compound, and grown under 16 h light/8 h dark cycle at 22°C in a growth chamber for 4 days (total 6‐day‐old). Then, root length of each seedling was measured. Images were taken with an E‐520 digital camera (Olympus Corp., Japan), and root length was measured using Image J 1.45S software (http://imageJ.net/Welcome). (*n* = 16, values represent mean ± SD.)

### 4.8 Measurement of Fresh Weights and Accumulated Anthocyanin

2‐day‐old seedlings of Col‐0 were transferred on 1/2 MS plate in the presence or absence of 1–10 µM of each compound, and grown under 16 h light/8 h dark cycle at 22°C in growth chamber for 4 days. 16 seedlings were cut at the base of the leaf, divided 4 seedlings into 4 pools, and weighed. For each pool, seedlings were homogenized with 100 µL aqueous methanol (0.1 % HCl in 50 % methanol/sterilized water (v/v)), and then incubated in dark at 4°C for 3 h. After incubation, added 100 µL chloroform for the samples and vortex for 3 s, then centrifuged at 15 000× *g* (4°C) for 2 min and collected aqueous phase. Total anthocyanins were determined by measuring the A530 and A657 using a spectrophotometer (NanoPhotometerN60, IMPLEN) and the content was calculated from A530‐0.25 × A657 per fresh weight (mg). (*n* = 4, values represent mean ± SD.)

### 4.9 Surface Plasmon Resonance Analysis

Surface plasmon resonance experiments were performed using a Biacore T200 instrument (Cytiva, Sweden). MBP‐JAZ1‐His_6_ and the control protein b2‐microglobulin (b2m) were covalently immobilized on CM5 chips (Cytiva, Sweden) using the amine coupling method. COI1 (0.24 µM) and JA‐Ile (10 µM) were pre‐mixed under the conditions of Biacore running buffer; 2.2% DMSO in HBS‐P^+^ (10 mM HEPES (pH 7.4), 150 mM NaCl, and 0.005% Surfactant P20, Cytiva, Sweden). Subsequently, **5** was incubated in this solution for 20 min. A triple mixture was injected onto the immobilized MBP‐JAZ1‐His_6_ chip, and the binding response at each concentration (from 0.09375 to 6 µM for **5**) was calculated by subtracting the response of the MBP‐JAZ1‐His_6_ flow cell from the equilibrium response measured in the control flow cell. Kinetic constants were derived by fitting rate equations, based on the simple 1:1 Langmuir binding model (A+B⇄AB), using the curve fitting facility of the Biacore T200 Evaluation Software (version 1.0, Cytiva, Sweden). A strong correlation is observed between the *K*_D_ measured by SPR and the *K*_i_ [37]. The conversion between *K*_D_ and *K*_i_ values is estimated by determining the inhibitory RU due to the concentration‐dependent binding of **5** to COI1 within the COI1 and JA‐Ile complex, followed by fitting to a 1:1 Langmuir binding model, leading to the estimation of *K*_D_ = *K*_i_ in the case of competitive inhibition [38].

### 4.10 Statistical Analyses

Statistical analysis was performed using CoStat (version 6.400, CoHort Software, USA). Data are presented as the mean ± standard deviation (SD). An analysis of variance (ANOVA) was used for data analysis. Different letters within a column indicate statistically significant differences (*p* < 0.05) by the Tukey HSD *post hoc* test.

## Funding

This work was financially supported by Grants‐in‐Aid for Scientific Research from Japan Society for the Promotion of Science (JSPS), Japan (Nos. 23H00316, 23K17967, 22KK0076, 21K19037, 20H00402, JPJSBP120229905, and JPJSBP120239903 to MU) and a Grant‐in‐Aid for Transformative Research Areas (A) “Latent Chemical Space” [JP23H04880 and JP23H04883] for MU from the Ministry of Education, Culture, Sports, Science and Technology, Japan. This research was also supported by Discovery (Basis for Supporting Innovative Drug Discovery and Life Science Research (BINDS)) from AMED under Grant Number JP25ama121037 to KM, and PID2019‐107012RB‐100 funded by MICIU/AEI/10.13039/501100011033 and EU/FEDER to RT and AC, and Grant CEX2023‐001386‐S funded by MICIU/AEI/ 10.13039/501100011033.

## Conflicts of Interest

The authors declare no conflicts of interest.

## Supporting information




**Supporting File**: advs73672‐sup‐0001‐SuppMat.pdf.

## Data Availability

The data that support the findings of this study are available in the supplementary material of this article.
