# Supplementary material for: A Reactive Antagonist Strategy: Cysteine‐Directed Covalent Molecular Glue in Plant Hormone Receptor Regulation
Source: Adv Sci (Weinh). 2026 Jan 8;13(10):e17463. doi: 10.1002/advs.202517463 (PMC12915133; doi:10.1002/advs.202517463)
Supplement: Supplementary file 1 — Supporting File: advs73672‐sup‐0001‐SuppMat.pdf. [file ADVS-13-e17463-s001.pdf]

WILEY-VCH

Supporting Information

A Reactive Antagonist Strategy: Cysteine-directed Covalent Molecular Glue in Plant Hormone Receptor Regulation

Minoru Ueda, Kotaro Matsumoto, Takao Nomura, Yousuke Takaoka, Taichi Okumura, Wataru Kozaki, Hikaru Hoshikawa, Yuho Nishizato, Andrea Chini, Roberto Solano, Katsumi Maenaka*

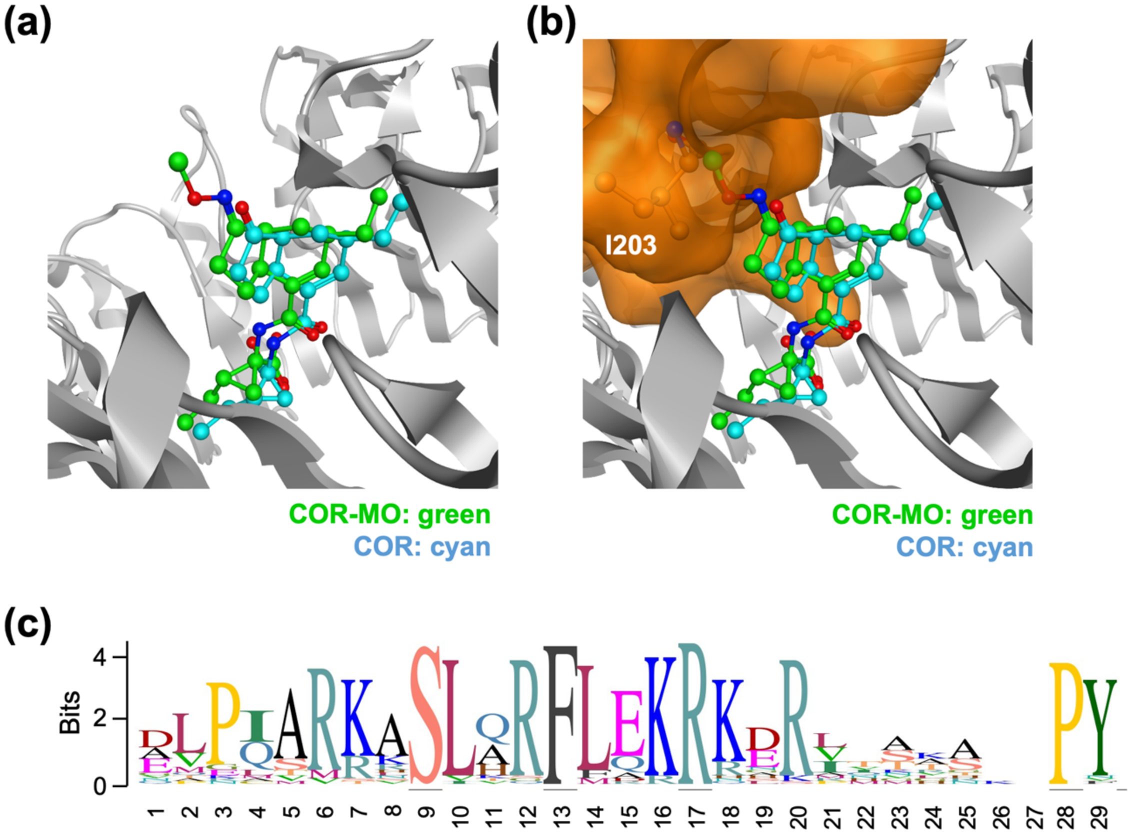

Figure S1. (a) Overlay of the docked COR-MO (green) complexed with COI1^{apo} and the reported crystal structure of **2** (cyan)/COI1/JAZ1. **(b)** A highlight image showing the position of I203 of JAZ1 in the crystal structure of COI1/**2**/JAZ1 superimposed on **(a)**. **(c)** Sequence logos of the Jas motif of JAZs in various species shown in **(d)**. **(d)** Each sequence of Jas motifs: 98 JAZs in 10 plant species are aligned. Arrow indicates position which is aligned to Ile203 of AtJAZ1 (shown in the next page).

(d)

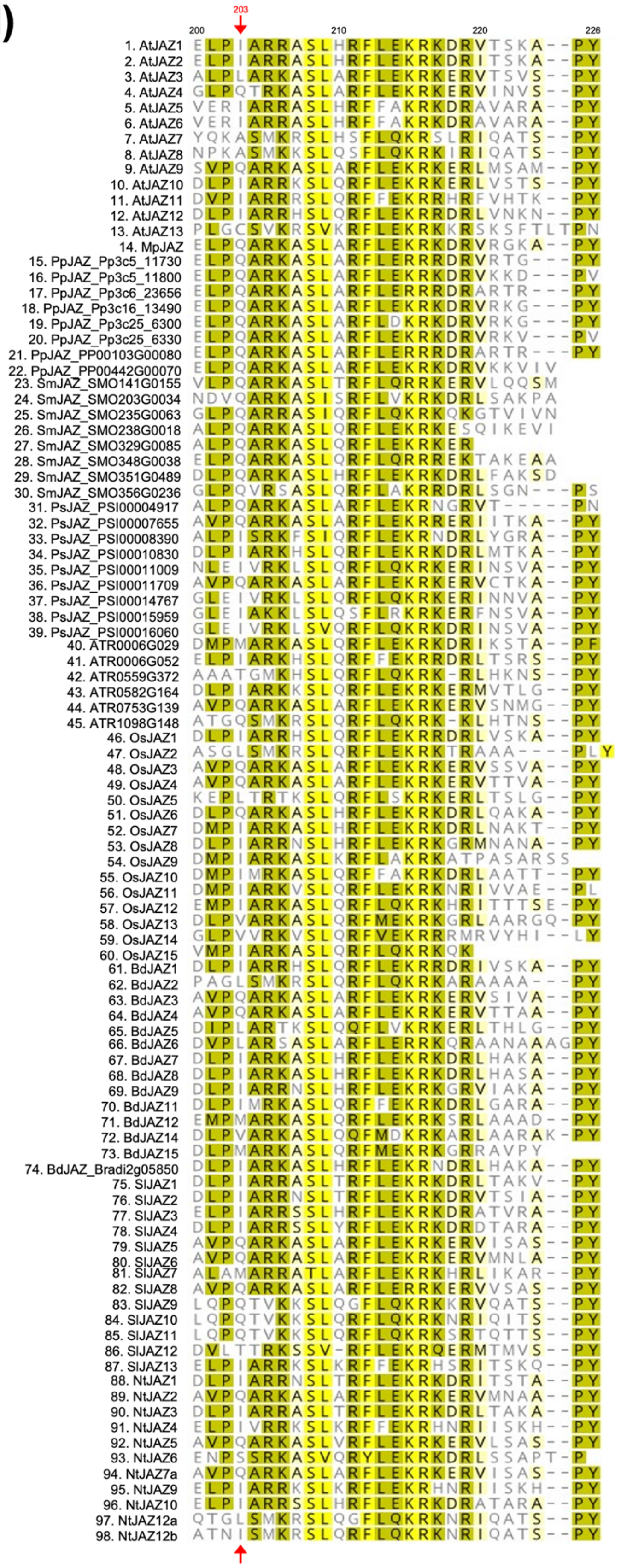
203
200
210
220
226
1. AtJAZ1
2. AtJAZ2
3. AtJAZ3
4. AtJAZ4
5. AtJAZ5
6. AtJAZ6
7. AtJAZ7
8. AtJAZ8
9. AtJAZ9
10. AtJAZ10
11. AtJAZ11
12. AtJAZ12
13. AtJAZ13
14. MpJAZ
15. PpJAZ_Pp3c5_11730
16. PpJAZ_Pp3c5_11800
17. PpJAZ_Pp3c6_23656
18. PpJAZ_Pp3c16_13490
19. PpJAZ_Pp3c25_6300
20. PpJAZ_Pp3c25_6330
21. PpJAZ_PP00103G00080
22. PpJAZ_PP00442G00070
23. SmJAZ_SMO141G0155
24. SmJAZ_SMO203G0034
25. SmJAZ_SMO235G0063
26. SmJAZ_SMO238G0018
27. SmJAZ_SMO329G0085
28. SmJAZ_SMO348G0038
29. SmJAZ_SMO351G0489
30. SmJAZ_SMO356G0236
31. PsJAZ_PSI00004917
32. PsJAZ_PSI00007655
33. PsJAZ_PSI00008390
34. PsJAZ_PSI00010830
35. PsJAZ_PSI00011009
36. PsJAZ_PSI00011709
37. PsJAZ_PSI00014767
38. PsJAZ_PSI00015959
39. PsJAZ_PSI00016060
40. ATR0006G029
41. ATR0006G052
42. ATR0559G372
43. ATR0582G164
44. ATR0753G139
45. ATR1098G148
46. OsJAZ1
47. OsJAZ2
48. OsJAZ3
49. OsJAZ4
50. OsJAZ5
51. OsJAZ6
52. OsJAZ7
53. OsJAZ8
54. OsJAZ9
55. OsJAZ10
56. OsJAZ11
57. OsJAZ12
58. OsJAZ13
59. OsJAZ14
60. OsJAZ15
61. BdJAZ1
62. BdJAZ2
63. BdJAZ3
64. BdJAZ4
65. BdJAZ5
66. BdJAZ6
67. BdJAZ7
68. BdJAZ8
69. BdJAZ9
70. BdJAZ11
71. BdJAZ12
72. BdJAZ14
73. BdJAZ15
74. BdJAZ_Bradi2g05850
75. SlJAZ1
76. SlJAZ2
77. SlJAZ3
78. SlJAZ4
79. SlJAZ5
80. SlJAZ6
81. SlJAZ7
82. SlJAZ8
83. SlJAZ9
84. SlJAZ10
85. SlJAZ11
86. SlJAZ12
87. SlJAZ13
88. NtJAZ1
89. NtJAZ2
90. NtJAZ3
91. NtJAZ4
92. NtJAZ5
93. NtJAZ6
94. NtJAZ7a
95. NtJAZ9
96. NtJAZ10
97. NtJAZ12a
98. NtJAZ12b

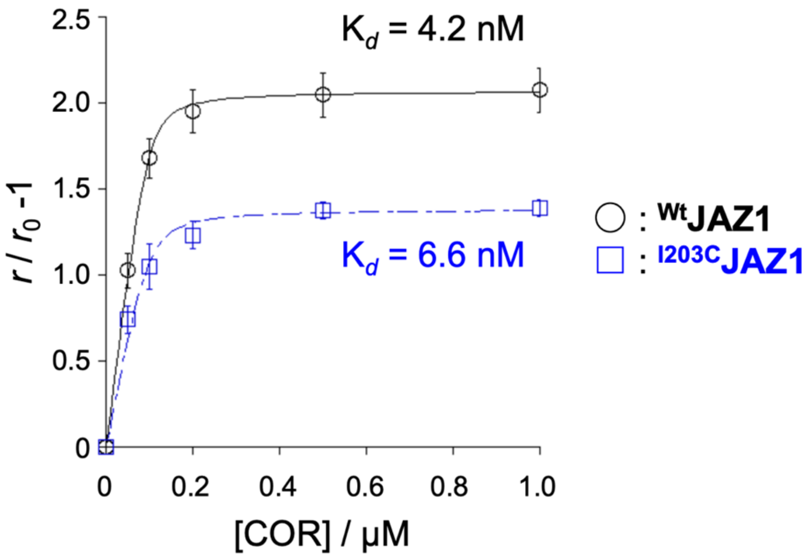

Figure S2. Fluorescence anisotropy changes of Fl-^{wt}JAZ1P (black circle) or Fl-^{I203C}JAZ1P (blue square) with GST-COI1 upon addition of COR (**2**, 0–1 μM). Results shown are mean with s.d.

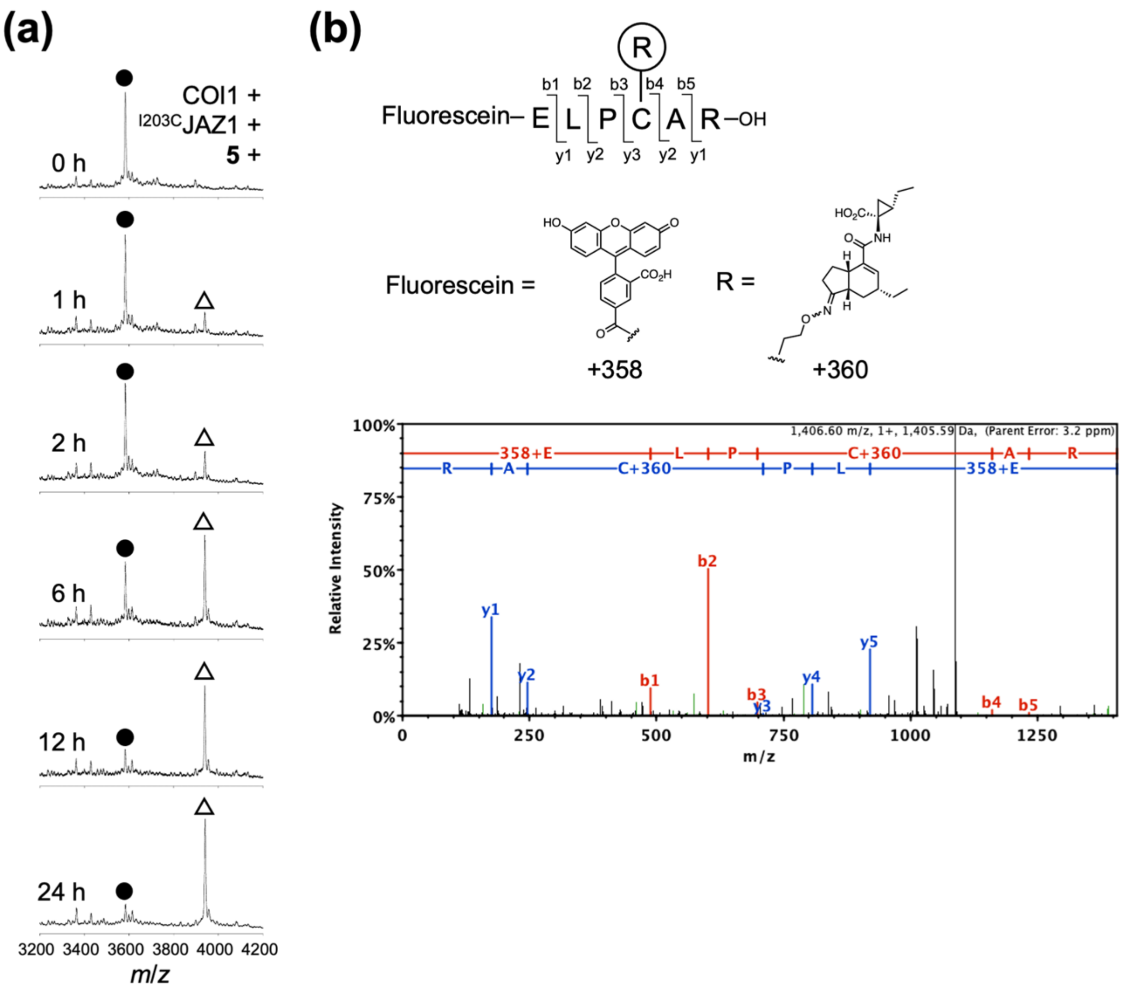

Figure S3. Characterization of the covalent modification of ^{I203C}JAZ1P with **5** and COI1. **(a)** Time trace of the *in vitro* labeling experiments of Fl-^{I203C}JAZ1P (1 μM) with GST-COI1 (1 μM) and **5** (1 μM) (0–24 h). **(b)** MS/MS analyses of the labeled Fl-^{I203C}JAZ1P with **5** and GST-COI1.

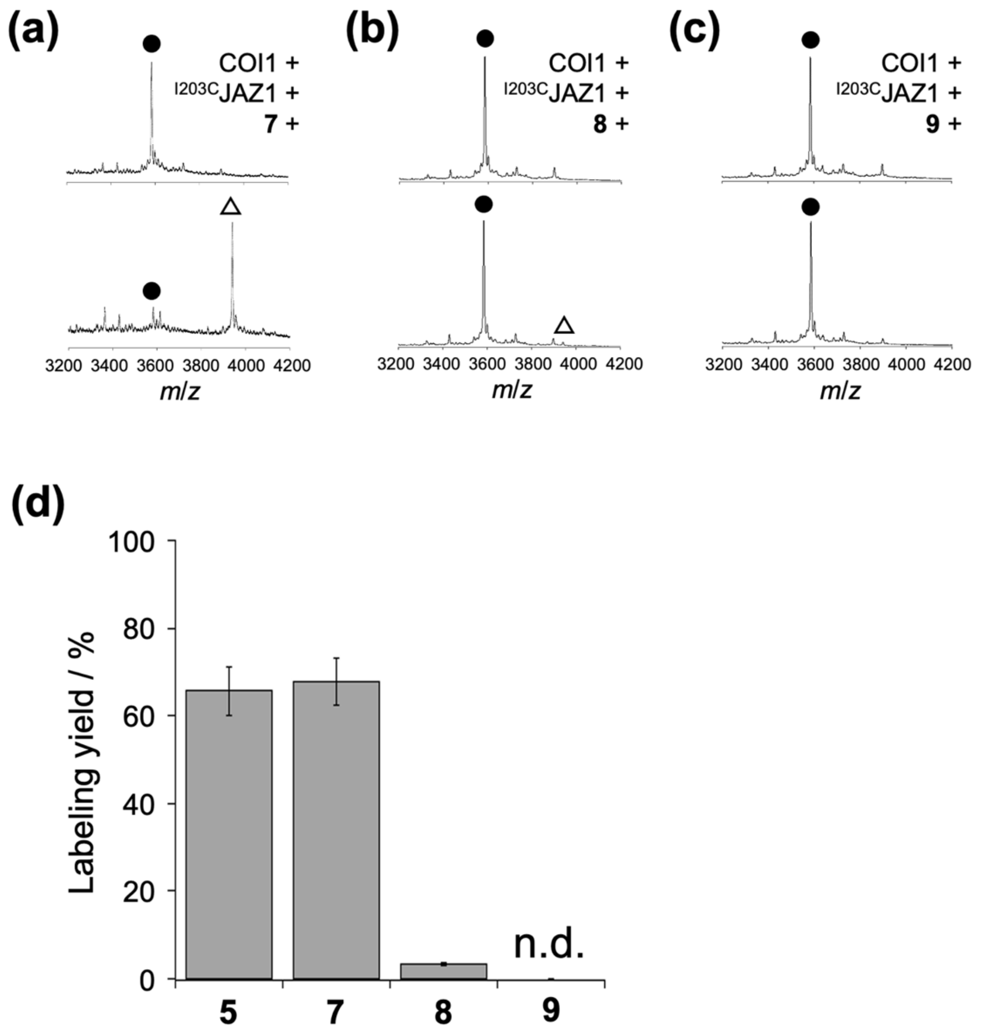

Figure S4. (a-c) MALDI-TOF MS analyses of the *in vitro* labeling of Fl-^{I203C}JAZ1P (1 μM) with GST-COI1 (1 μM) and the reactive antagonists (1 μM); **(a) 7**, **(b) 8**, **(c) 9**. The reaction mixtures were incubated for 0 h (top) or 12 h (bottom) at 22 ºC. **(d)** Labeling yields of Fl-^{I203C}JAZ1P with GST-COI1 and **5**, **7**, **8** and **9**. Results shown are mean with s.d.

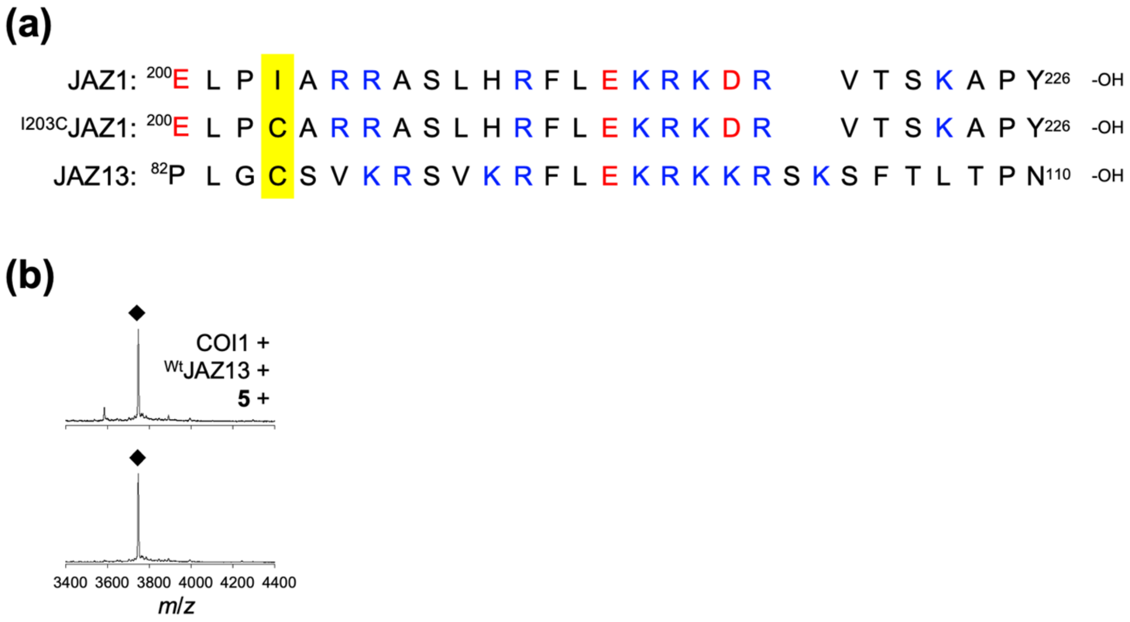

Figure S5. **(a)** Sequence alignment of JAZ1, [I203C]JAZ1 and JAZ13. The position which is correspond to Ile203 in JAZ1 are highlighted. **(b)** MALDI-TOF MS analyses of the in vitro labeling of Fl-[wt]JAZ13P (1 µM) with GST-COI1 (1 µM) and **5** (1 µM). The reaction mixtures were incubated for 0 h (top) or 12 h (bottom) at 22 ºC.

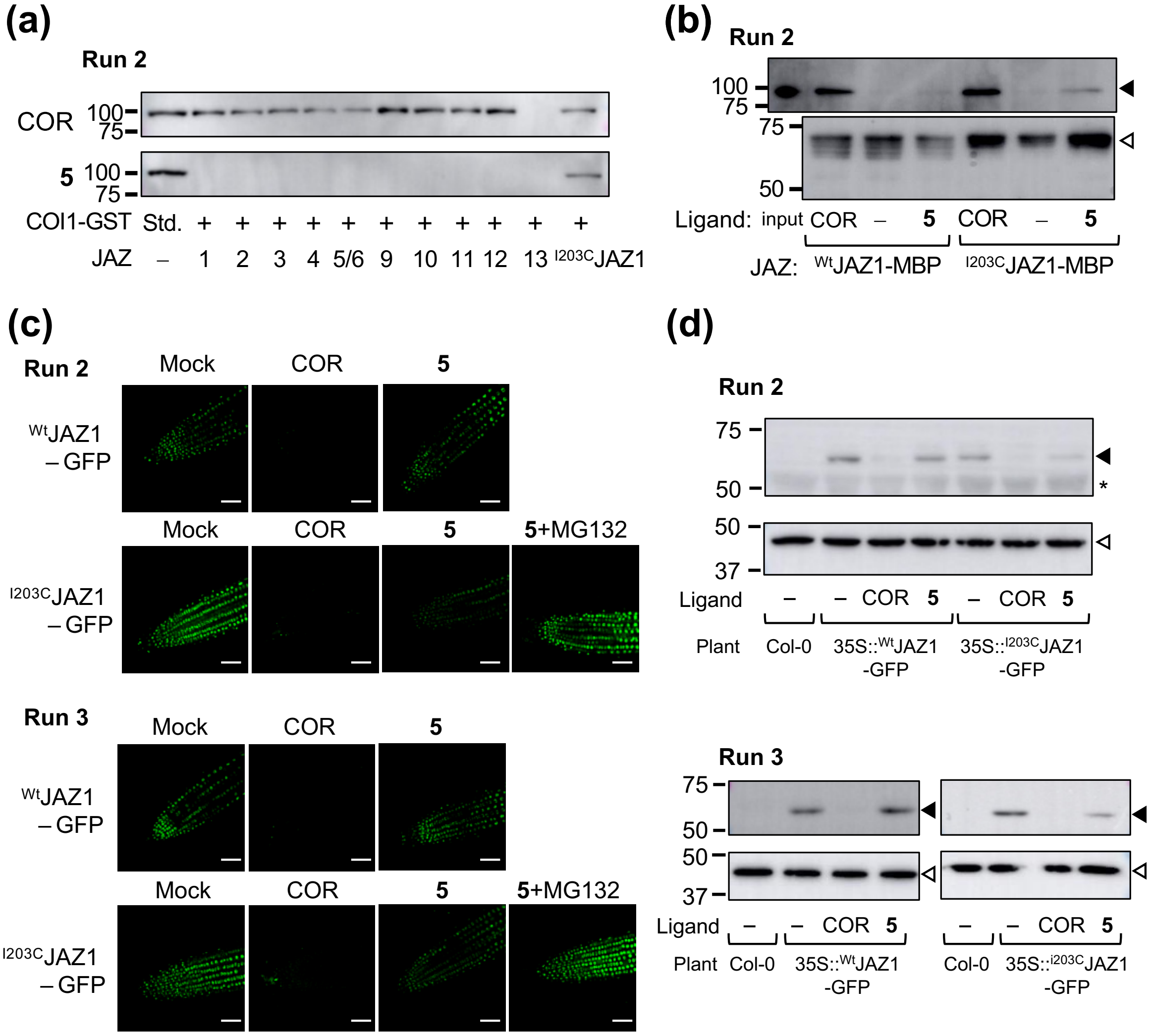

Figure S6. (a) Pull-down assay of GST-COI1 (5 nM) with each Fl-JAZP (10 nM) in the presence of **2** (COR, 100 nM, top) or **5** (100 nM, bottom) (reproducibility confirmation of the results shown in **Figure 2g**). Anti-Fl antibody was used for immunoprecipitation and anti-GST-HRP was used for chemiluminescence detection of GST-COI1. **(b)** Pull-down assay of GST-COI1 (5 nM) with full-length JAZ1-MBP (40 nM, ^{wt}JAZ1-MBP or ^{I203C}JAZ1-MBP) in the absence of presence of **2** (COR, 100 nM) or **5** (100 nM). Amylose resin was used for pull-down. Amylose resin was used for pull-down. Anti-GST-HRP was used for chemiluminescence detection of GST-COI1 (black triangles). Anti-MBP antibody and rat-IgG-HRP were used to visualize JAZ1-MBP protein levels as the input materials (white triangles). (reproducibility confirmation of the results shown in **Figure 2h**). **(c)** Fluorescence live imaging of 35S::JAZ1-GFP treated with mock, **2** (COR, 1 µM), or **5** (1 µM) in the absence or presence of MG132 (100 µM) for 2 h at 22 ºC; top: 35S::^{wt}JAZ1-GFP, bottom 35S::^{I203C}JAZ1-GFP (reproducibility confirmation of the results shown in **Figure 2i**). **(d)** Western blotting analyses of 35S::JAZ1-GFP treated with mock, 2 (COR, 1 µM) or 5 (1 µM) for 2 h at 22 ºC. Anti-GFP-HRP (top, 56

kDa, black triangles) or anti-b-actin (bottom, 40 kDa, white triangles) was used for chemiluminescence detection (reproducibility confirmation of the results shown in **Figure 2j**).

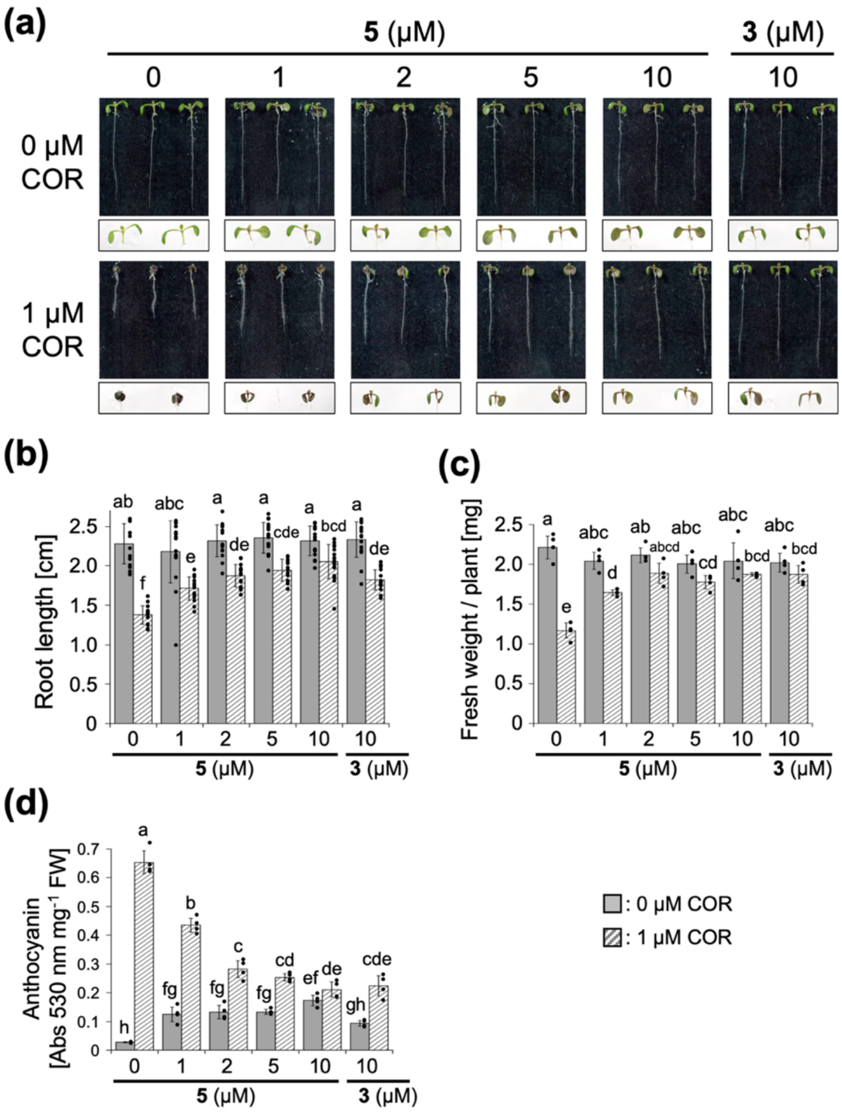

Figure S7. Antagonist activity of **5** or **3** in vivo. **(a)** *Arabidopsis* WT seedlings grown for 6 days on 1/2 MS medium containing **5** (0–10 µM) or COR-MO (**3**, 10 µM) in the absence or presence of COR (**2,** 1 µM). **(b-d)** Quantification of Root length **(b)**, fresh weight **(c)** and anthocyanin accumulation **(d)** of 6-d-old seedlings grown on 1/2 MS medium containing **5** (0–10 µM) or COR-MO (**3**, 10 µM) in the absence or presence of COR (**2,** 1 µM) (n = 16 for **(b)**, n = 4 for **(c)** and **(d)**). The results shown are the mean with s.d. The significant differences were evaluated by one-way ANOVA/Tukey HSD *post hoc* test. Different letters represent a significant difference at $p < 0.05$.

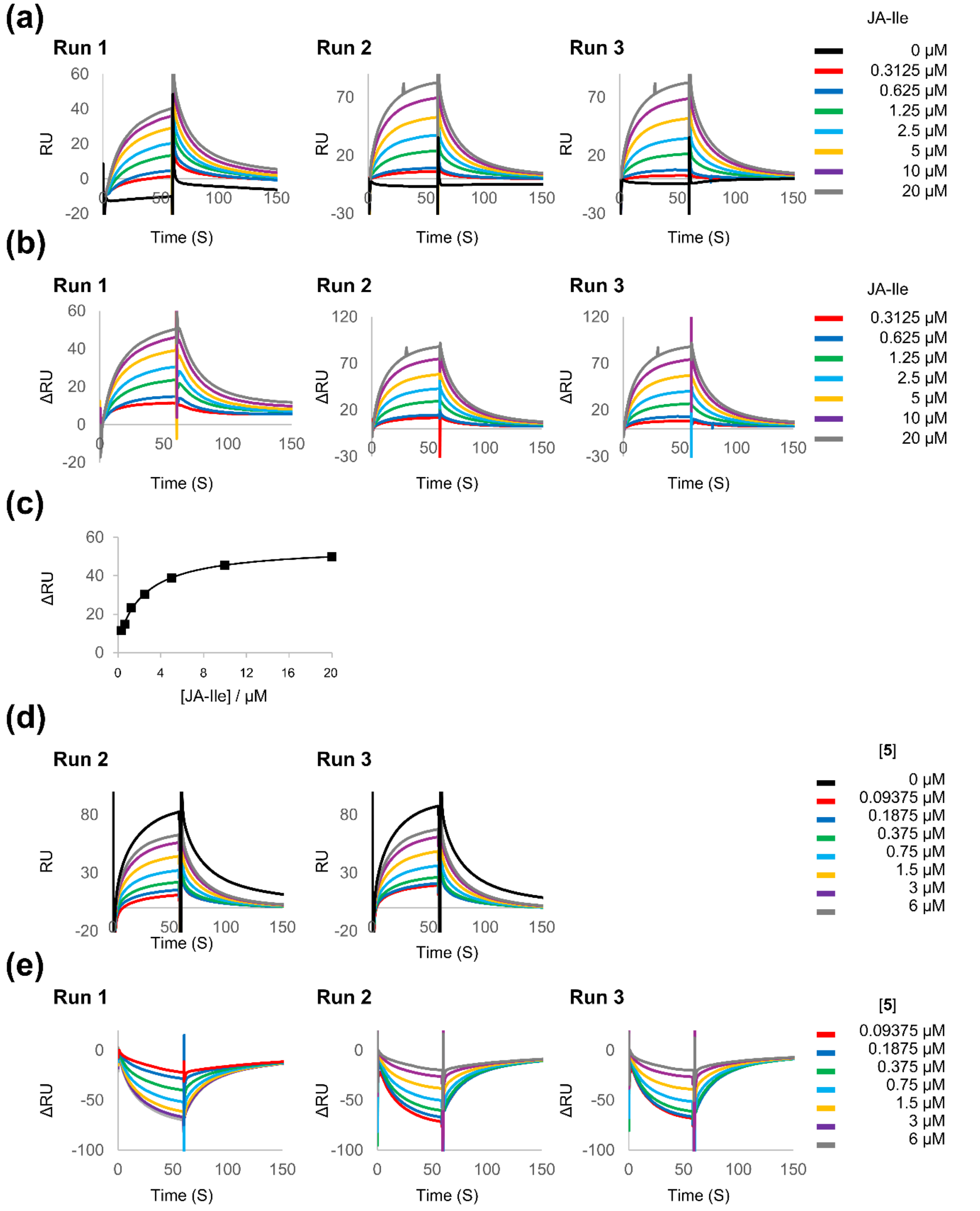

Figure S8. SPR assay using COI1 (20 μg/mL) and JAZ1 chip. SPR assays were repeated three times. (**a**) RU increase in dose-dependence of JA-Ile. (**b**) ΔRU of (**a**) with reference to JA-Ile (0 μM). (**c**) ΔRU dose-response curves of Run 1 in (**b**). (**d**) The additional two Runs of Fig. 3d. (**e**) ΔRU of Fig. 3d and (**d**) with reference to **5** (0 μM).

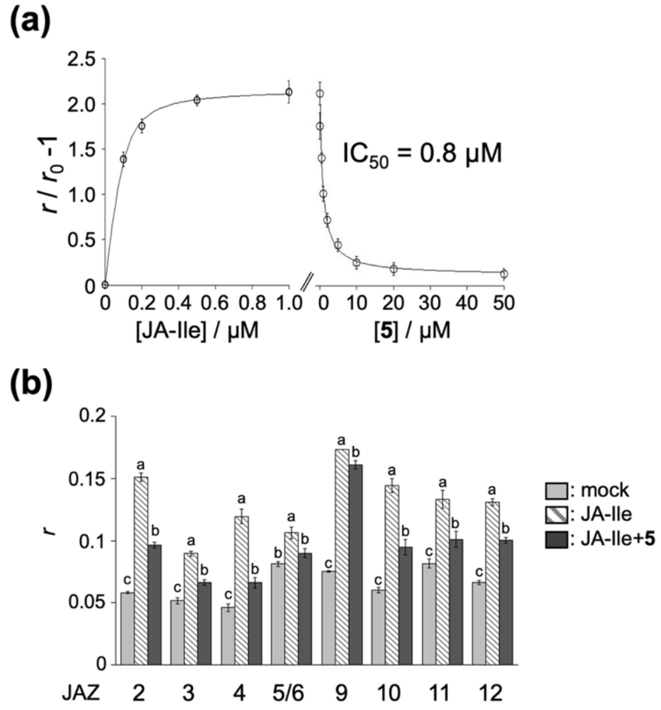

Figure S9. Antagonist activity of **5** *in vitro*. **(a)** Fluorescence anisotropy changes of Fl-JAZ1P with GST-COI1 upon addition of JA-Ile (**1**, 0–1 μM), followed by addition of **5** (0–50 μM) (n = 3). Results shown are mean with s.d. **(b)** Fluorescence anisotropy (*r*) values of Fl-AtJAZ2-6/9-12 with GST-COI1 in the absence or presence of JA-Ile (**1**, 1 μM) and **5** (10 μM) (n = 3). Results shown are mean with s.d. The significant differences were evaluated by one-way ANOVA/Tukey HSD *post hoc* test. Different letters represent a significant difference at $p < 0.05$.

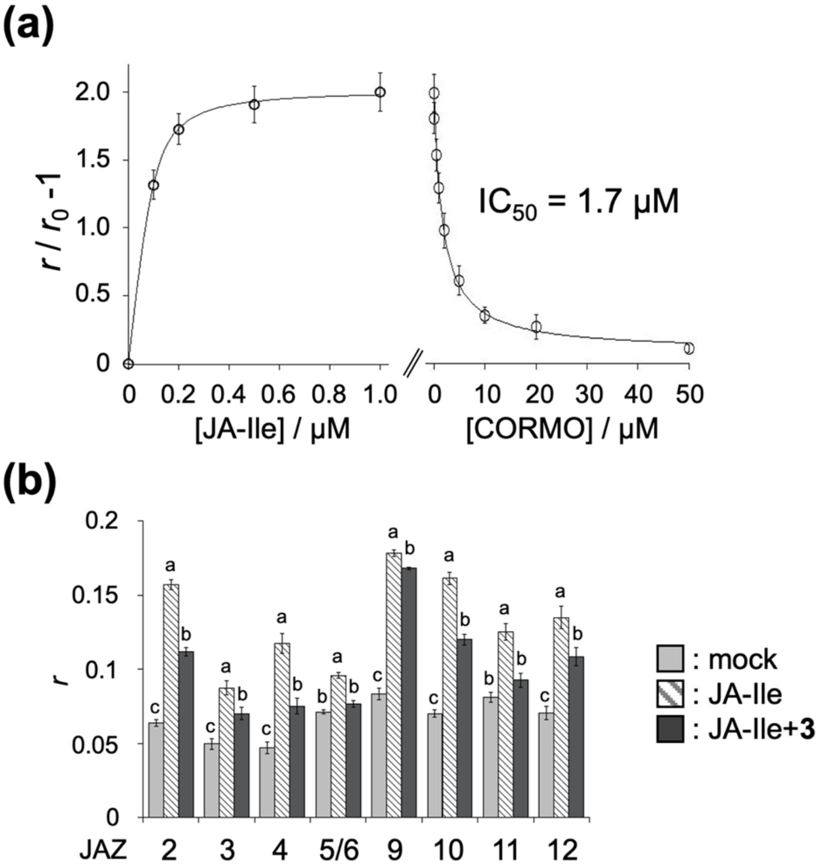

Figure S10. Antagonist activity of COR-MO (**3**) *in vitro*. **(a)** Fluorescence anisotropy changes of Fl-JAZ1P with GST-COI1 upon addition of JA-Ile (**1**, 0–1 μM), followed by addition of **3** (0–50 μM) (n = 3). Results shown are mean with s.d. **(b)** Fluorescence anisotropy (r) values of Fl-AtJAZ2-6/9-12 with GST-COI1 in the absence or presence of JA-Ile (**1**, 1 μM) and **3** (10 μM) (n = 3). Results shown are mean with s.d. The significant differences were evaluated by one-way ANOVA/Tukey HSD *post hoc* test. Different letters represent a significant difference at $p < 0.05$.

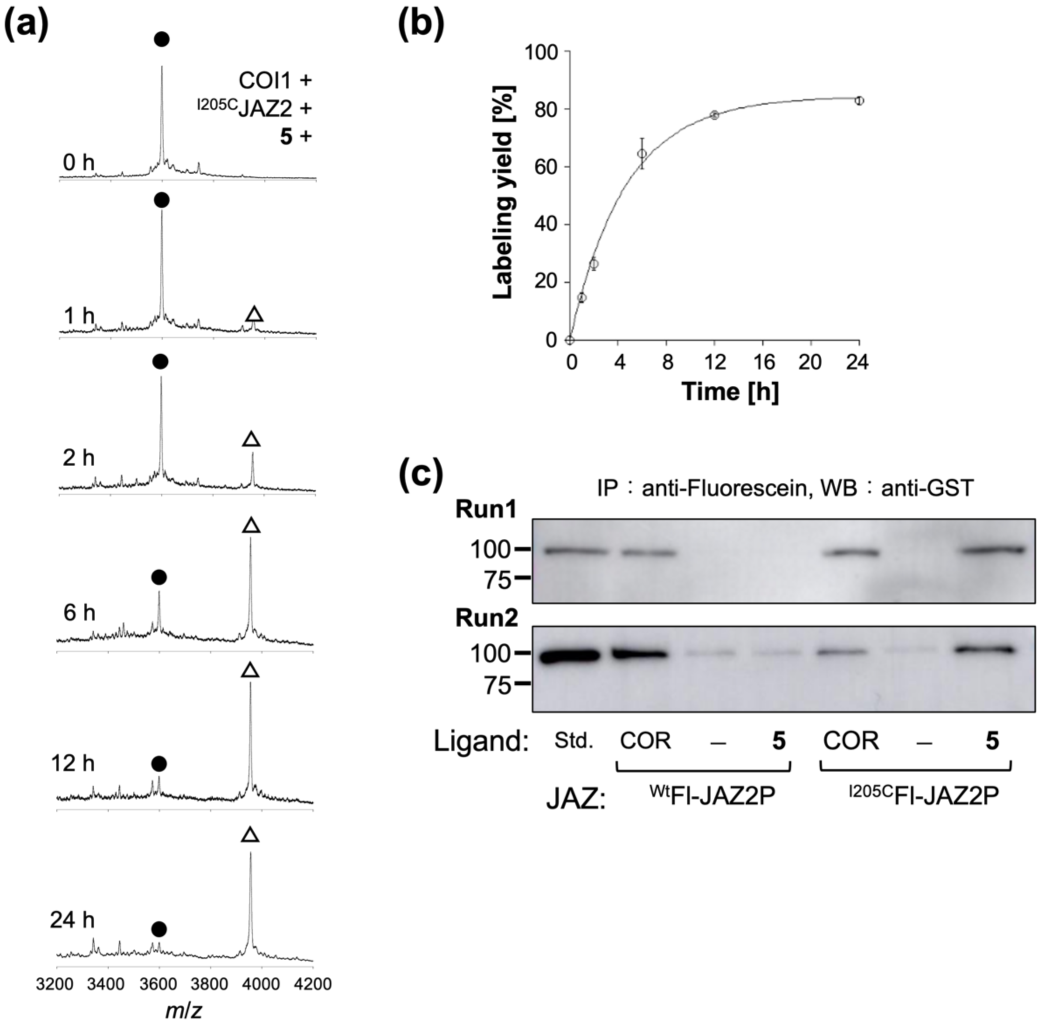

Figure S11. (a) Time trace of the *in vitro* labeling experiments of Fl-^{I205C}JAZ2P (1 μM) with GST-COI1 (1 μM) and **5** (1 μM) (0–24 h). **(b)** The time dependency of the labeling yields of Fl-^{I205C}JAZ2P (1 μM) with GST-COI1 (1 μM) and **5** (1 μM), monitored by MALDI-TOF MS. Results shown are mean with s.d. **(c)** Pull-down assay of GST-COI1 (5 nM) with Fl-JAZ2P (10 nM, Fl-^{wt}JAZ2P or Fl-^{I205C}JAZ2P) in the absence of presence of **2** (COR, 100 nM) or **5** (100 nM). Anti-Fl antibody was used for immunoprecipitation and anti-GST-HRP was used for chemiluminescence detection of GST-COI1. The experiments were repeated twice with similar results (shown as Run1 and Run2).

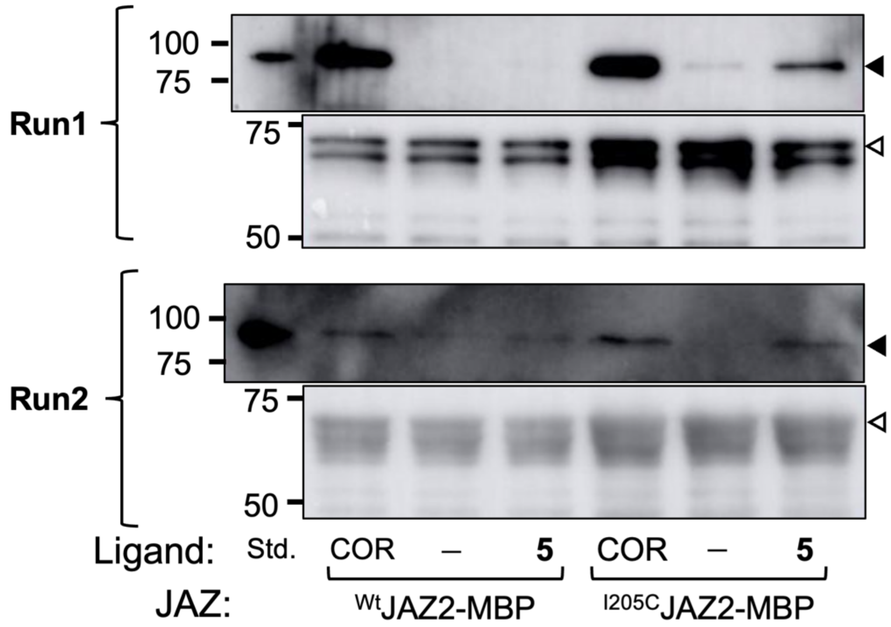

Figure S12. Pull-down assay of GST-COI1 (5 nM) with full-length JAZ2-MBP (40 nM, ^{wt}JAZ2-MBP or ^{I205C}JAZ2-MBP) in the absence of presence of **2** (COR, 100 nM) or **5** (100 nM). Amylose resin was used for pull-down. Anti-GST-HRP was used for chemiluminescence detection of GST-COI1 (black triangles). Anti-MBP antibody and rat-IgG-HRP were used to visualize JAZ2-MBP protein levels as the input materials (white triangles). The experiments were repeated twice with similar results (shown as Run1 and Run2).

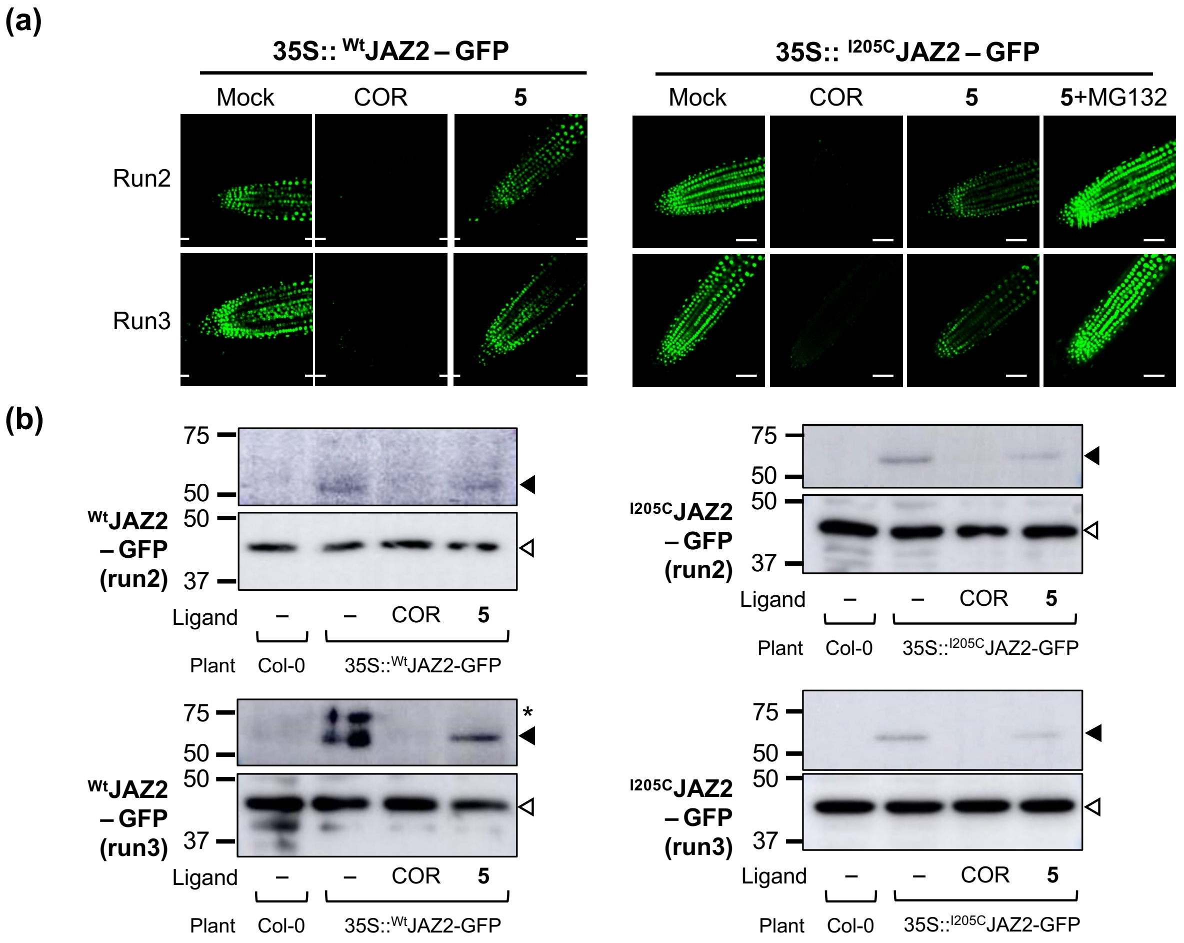

Figure S13. Additional duplicate experiments shown in **Figure 4bc**. **(a)** Fluorescence live imaging of 35S::JAZ2-GFP treated with mock, **2** (COR, 1 µM), or **5** (1 µM) in the absence or presence of MG132 (100 µM) for 2 h at 22 ºC; left: 35S::^{wt}JAZ2-GFP, right: 35S::^{I205C}JAZ2-GFP. **(b)** Western blotting analyses of 35S::JAZ2-GFP treated with mock, **2** (COR, 1 µM) or **5** (1 µM) for 2 h at 22 ºC. Anti-GFP-HRP (top, 56 kDa, black triangles) or anti-b-actin (bottom, 40 kDa, white triangles) was used for chemiluminescence detection. Asterisk indicates non-specific protein which was detected with anti-GFP antibody.

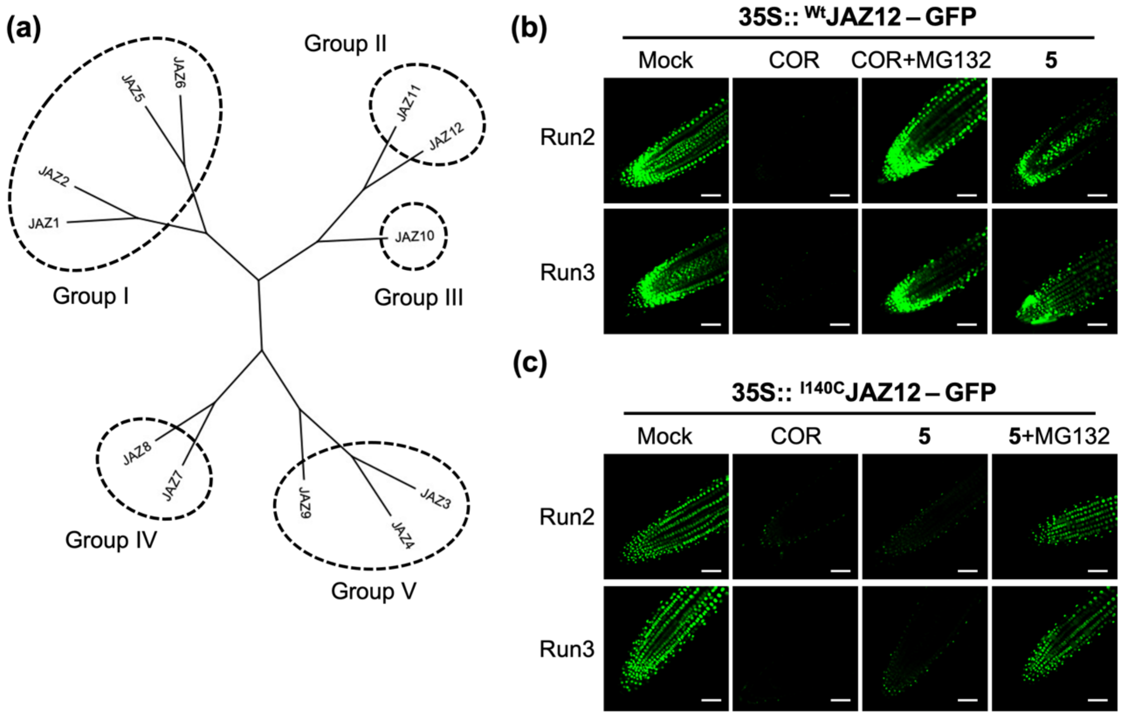

Figure S14. (a) Phylogenetic tree of all 12 JAZ proteins. The full length amino acids of JAZ proteins were aligned with MUSCLE (Edgar, R. C., *Nucleic Acids Res.* 32, 1792-1707 (2004)). The phylogenetic tree was generated with the Neighbor-joining method using Geneious Prime (www.geneious.com). **(b, c)** Additional duplicate experiments shown in **Figure 4d**. Fluorescence live imaging of 35S::^{wt}JAZ2-GFP (**b**) or 35S::^{I140C}JAZ2-GFP (**c**) treated with mock, **2** (COR, 1 μM) or **5** (1 μM) in the absence or presence of MG132 (100 μM) for 2 h at 22 ºC.

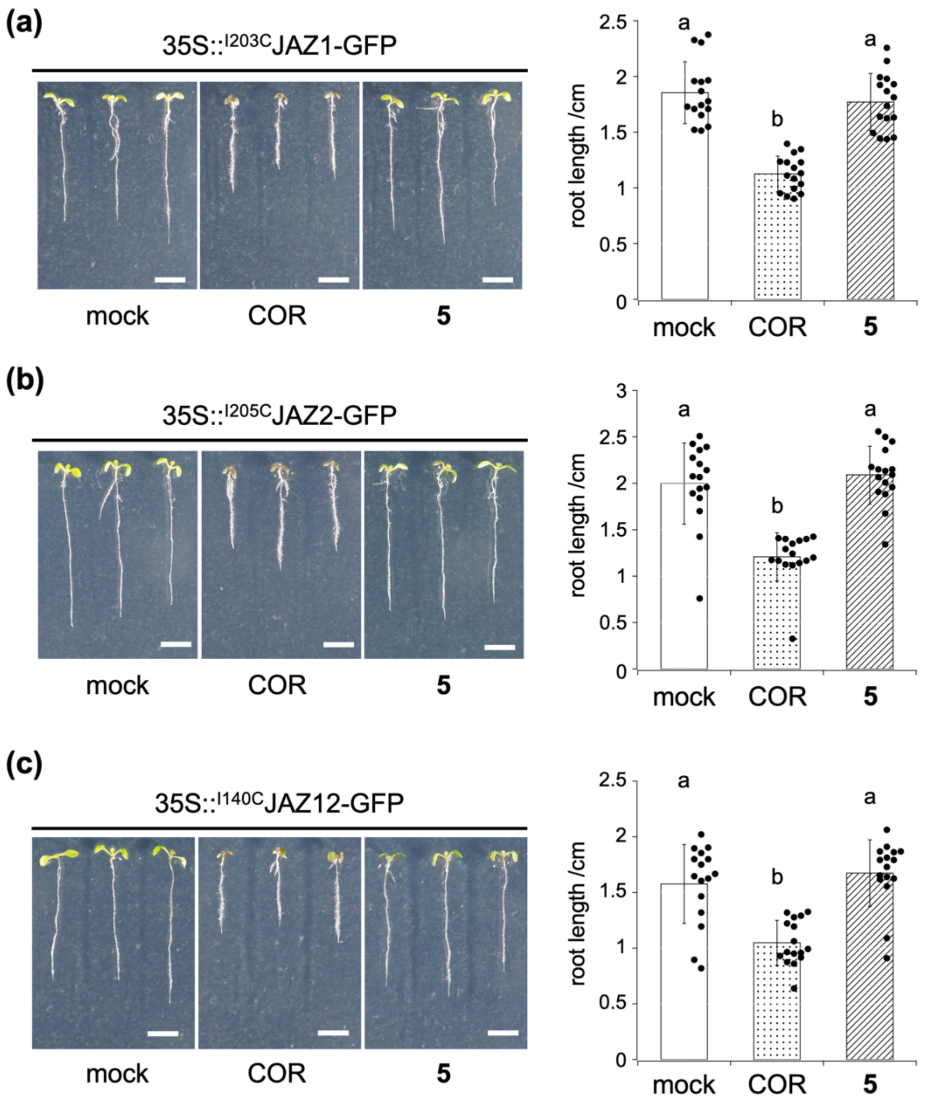

Figure S15. The representative images of *Arabidopsis* 35S::^{I203C}JAZ1-GFP (**a**, left), 35S::^{I205C}JAZ2-GFP (**b**, left), or 35S::^{I140C}JAZ12-GFP (**c**, left) seedlings grown for 6 days on 1/2 MS medium in the absence (mock) and presence of COR (**2**, 1 µM) or **5** (1 µM). Scale bar 0.5 cm. Root length inhibition rate of these seedlings 35S::^{I203C}JAZ1-GFP (**a**, right), 35S::^{I205C}JAZ2-GFP (**b**, right), or 35S::^{I140C}JAZ12-GFP (**c**, right) grown on 1/2 MS medium in the absence (mock) and presence of COR (**2**, 1 µM) or **5** (1 µM) shown in each image (n = 16). Results shown are the mean with s.d. The significant differences were evaluated by one-way ANOVA/Tukey HSD *post hoc* test. Different letters represent a significant difference at $p < 0.05$.

The experiments were repeated three times with similar results.

Table S1. Primers used in this study

No.	Name	Sequence (5' to 3')
1	*pGWB5_35S_SalI_F*	CACCGTCGACAAGCTTGCATGCCTGCAGGTCC
2	*pGWB5_GFP_XhoI_R*	GCGGCTCGAGCTCGGGCCGCTTTACTTGTAC
3	*pGWB4_Adaptor_F*	AGCTTGTCGACGAATTCCTCGAGCT
4	*pGWB4_Adaptor_R*	CGAGGAATTCGTCGACA
5	*I205C_JAZ2_F_1*	CCCGTGCGCAAGAAGAGCTTCACTTCATCGGTTC
6	*I205C_JAZ2_R_1*	AGTTCGCAGGCTAAAGAGGCCGGGTTAGGCTG
7	*attB1_JAZ1_F*	GGGGACAAGTTTGTACAAAAAAGCAGGCTTCATGTCG AGTTCTATGGAATG
8	*attB2_JAZ1_R*	GGGGACCACTTTGTACAAGAAAGCTGGGTGTATTTCA GCTGCTAAACCGAG
9	*attB1_JAZ12_F*	GGGGACAAGTTTGTACAAAAAAGCAGGCTTCATGACTA AGGTGAAAGATGAG
10	*attB2_JAZ12_R*	GGGGACCACTTTGTACAAGAAAGCTGGGTGAGCAGTT GGAAATTCCTCCTTG
11	*I203CJAZ1_F*	CCTTGCGCTAGAAGAGCTTCAC
12	*I203CJAZ1_R*	AAGTTCTGTCAATGGTGTTGGGGA
13	*I140CJAZ12_F*	GCAAGGAGGCATTCGCTTCAACGATTCCTCG
14	*I140CJAZ12_R*	GCAAGGTAGATCAGCCGTGGATCTGCAAAACG
15	*pGWB_attB1_R2_F*	CTTGTACAAAGTGGTTCGATCTAGAGGATCCATGG
16	*pGWB_attB1_R1_R*	TTTGTACAAACTTGTTTGATTAGAGTCCCCCGTG
17	*pGWB_before35S_F2nd*	TGATTACGCCAAGCTTGCATGCCTGCAGG
18	*pGWB_afterGFP_R2nd*	GATCGGGGAAATTCGAGCTCGGGCCGCTTTAC

Synthesis of hydroxylamine

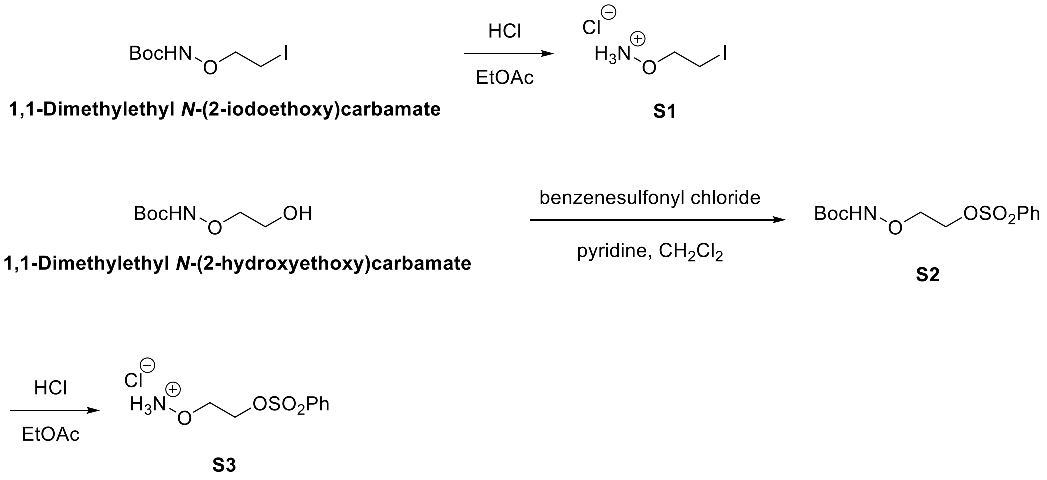

Synthesis of oxime

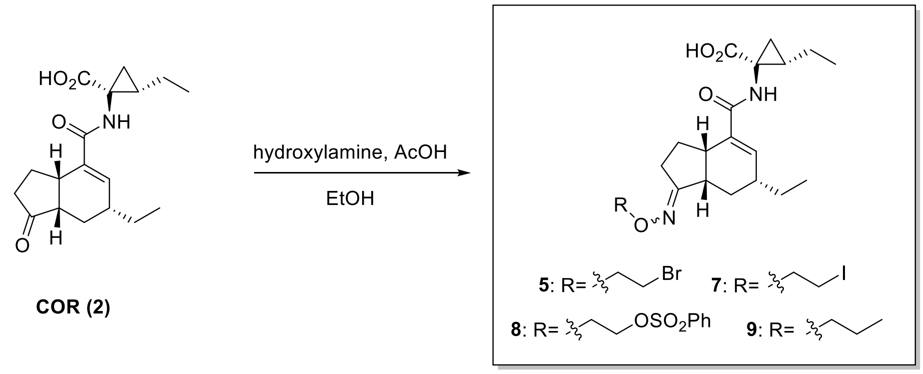

Scheme S1. Synthetic route of designed RAs.

Supplementary Methods

General materials and methods: All chemical reagents and solvents were obtained from commercial suppliers (Fujifilm Wako Pure Chemical Industries Co., Ltd., Nakalai Tesque Co., Ltd., Sigma-Aldrich Co., Ltd., Tokyo Chemical Industry Co., Ltd., Watanabe Chemical Industries Co., Ltd., Aldlab Chemicals LLC, Thermo Fisher Scientific K.K., GE Healthcare) and used without further purification. All anhydrous solvents were either dried by standard techniques and freshly distilled before use or purchased in anhydrous form and used as supplied. Reversed-phase high–performance liquid chromatography (HPLC) was carried out on a PU–4180 plus pump equipped with UV–4075 and MD–4010 detectors (JASCO, Tokyo, Japan). ^{1}H and ^{13}C NMR spectra were recorded on a JNM–ECS–400 spectrometer (JEOL, Tokyo, Japan). Chemical shifts are denoted in δ (ppm) relative to TMS or residual solvent peaks as internal standard (TMS, ^{1}H δ 0.00; $CDCl_3$, ^{13}C δ 77.0; CD_3OD, ^{1}H δ 3.31, ^{13}C δ 49.0; pyridine-*d5*, ^{1}H δ 8.74, ^{13}C δ 150.4). Fourier transforms infrared (FT/IR) spectra were recorded on an FT/IR–4100 (JASCO, Tokyo, Japan). High–resolution (HR) electrospray ionization (ESI)–mass spectrometry (MS) analyses were conducted using a microTOF II (Bruker Daltonics Inc., Billerica, MA, US). Optical rotations were measured using a JASCO P–2200 polarimeter (JASCO, Tokyo, Japan). Flash chromatography was performed on an Isolera system (Biotage Ltd., North Carolina, US). TLC analyses were performed on Silica gel F254 (0.25 mm or 0.5 mm, MERCK, Germany) or RP–18F254S (0.25 mm, MERCK). All reactions were carried out under air unless stated otherwise. Reverse-phase high-performance liquid chromatography (HPLC) was conducted on a PU-4180 plus with UV-4075 and MD-4010 detectors (JASCO Corp., Japan). UV detection was performed at 220 nm. MALDI-TOF/MS analysis was carried out on an autoflex (Bruker Daltonics Inc., MA). Ultraviolet (UV)-visible spectra were recorded on a UV-2600 spectrophotometer (JASCO Corp., Japan). For GUS quantification assay, fluorescence intensity and absorbance were measured on an Infinite 200 Pro spectrophotometer (TEKAN Co., Ltd., Switzerland). SDS-PAGE and western blotting were analyzed with a Mini-Protean III electrophoresis apparatus (Bio-Rad, Hercules, CA). Chemiluminescent signals were detected with a LAS 4000 imaging system (Fujifilm, Japan).

Synthesis and characterization of RA (5):

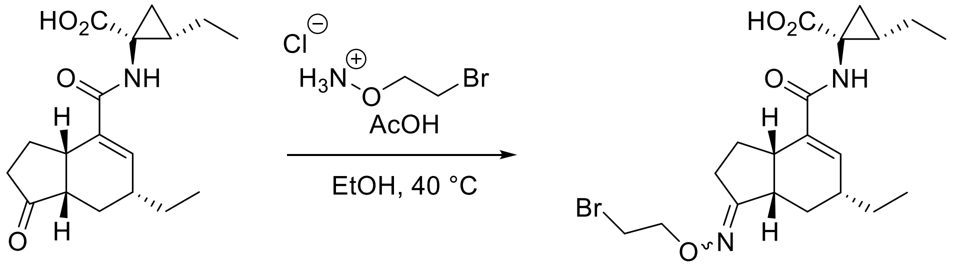

COR (2) **5**

COR (**2**) was prepared as previously described[S1,S2]. To a solution of **2** (9.0 mg, 28.2 μmol) in dehydrated EtOH (0.5 mL) was added 0.05 M AcOH in EtOH (1.13 mL, 56.4 μmol) and *O*-(2-bromoethyl)hydroxylamine hydrochloride[S3] (19.8 mg, 112 μmol). After being stirred at 40 ºC for 1.5 h, the reaction mixture was diluted with 50% aqueous MeCN and the mixture was purified HPLC equipped with COSMOSIL Cholester column (ϕ20 × 250 mm, Nacalai Tesque co., ltd.) with 0.05% TFA solution (CH_3CN / H_2O = 48 / 52) at flow rate of 10.0 mL/min to afford **5** (4.3 mg, 35% for major isomer; 2.2 mg, 17% for minor isomer) as a colorless oil. The major isomer was used for the following assay: ^{1}H NMR (400 MHz, $CDCl_3$, data for major isomer) δ_H: 6.37 (s, 1H), 6.33 (s, 1H), 4.30 (t, J = 6.4 Hz, 2H), 3.54 (t, J = 6.4 Hz, 2H), 2.89 (dt, J = 12.1, 6.1 Hz, 1H), 2.76-2.67 (m, 2H), 2.48-2.36 (m, 1H), 2.29 (dt, J = 12.0, 8.0 Hz, 1H), 2.17 (brs, 1H), 1.82 (dt, J = 13.2, 4.5 Hz, 1H), 1.68-1.32 (m, 7H), 1.28-1.24 (m, 1H), 1.14 (q, J = 12.5 Hz, 1H), 1.03 (t, J = 7.3 Hz, 3H), 0.98 (t, J = 7.4 Hz, 3H); ^{13}C NMR (100 MHz, $CDCl_3$) δ_C: 173.6, 170.8, 168.6, 138.6, 134.8, 72.9, 41.2, 37.9, 37.6, 33.7, 30.2, 29.7, 29.3, 28.1, 28.0, 27.0, 21.9, 20.9, 13.4, 11.3; HRMS (ESI, positive) m/z $[M+Na]^+$ Calcd for $C_{20}H_{29}N_2O_4BrNa$ 463.1203, Found 463.1200.

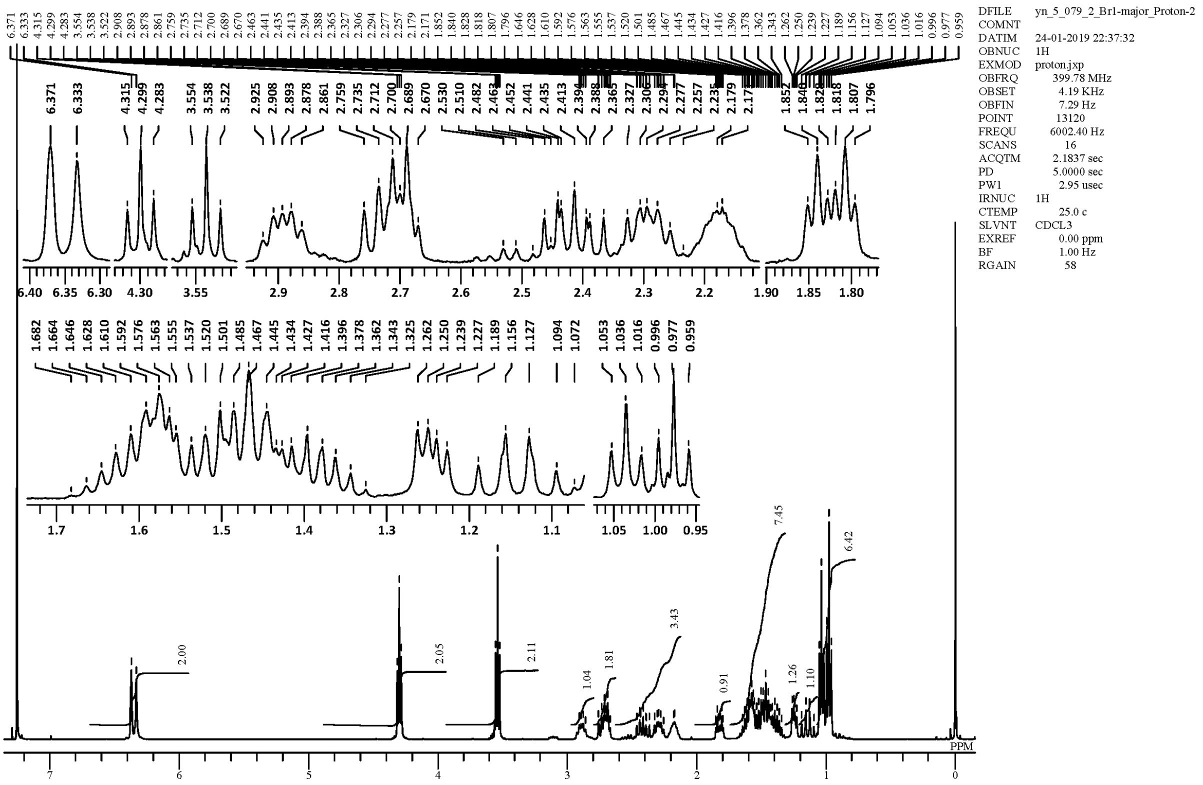

^{1}H NMR spectrum of compound **5**.

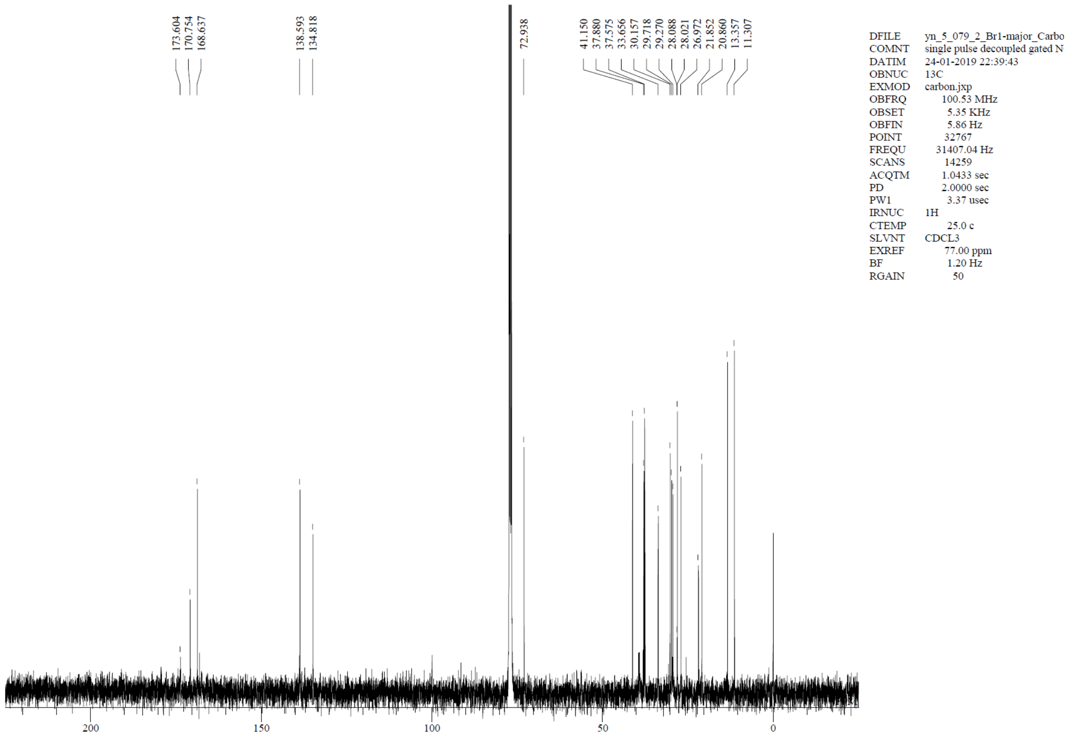

^{13}C NMR spectrum of compound **5**.

Synthesis of S1:

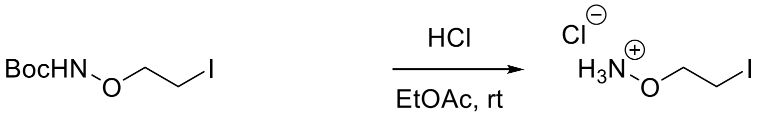

1,1-Dimethylethyl *N*-(2-iodoethoxy)carbamate **S1**

1,1-Dimethylethyl *N*-(2-iodoethoxy)carbamate (14.9 mg, 51.5 μmol) was treated with 4 M HCl in EtOAc (0.28 mL) at r.t. for 45 min. The mixture was concentrated *in vacuo* to afford crude **S1** (11.0 mg), and was used in the next step without further purification.

Synthesis and characterization of 7:

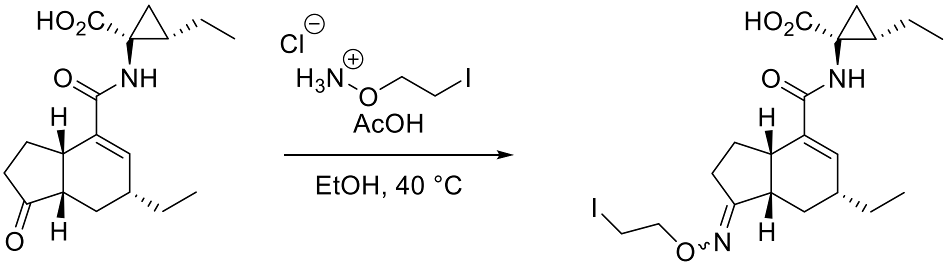

COR (2) **7**

COR (**2**) (2.8 mg, 8.7 μmol) and excess amount of crude **S1** (11.0 mg, ca. 49.2 μmol) were added to a solution of 0.05 M AcOH in EtOH (0.35 mL). The mixture was stirred at 40 °C for 1.5 h. Then, the mixture was concentrated *in vacuo* and the residue was purified by HPLC equipped with COSMOSIL Cholester column (ϕ20 × 250 mm, Nacalai Tesque co., ltd.) with 0.05% TFA solution (CH_3CN / H_2O = 48 / 52) at flow rate of 10.0 mL/min, the ratio of oxime isomers was about 2:1, Rt = 46 min as major isomer, Rt = 51 min as minor isomer) to afford **7** (2.3 mg, 63% for major isomer; 1.0 mg, 23% for minor isomer) as a colorless oil. The major isomer was used for the assays. major isomer: ^{1}H-NMR (400 MHz, $CDCl_3$) δ_H: 6.38 (s, 1H), 6.33 (s, 1H), 4.24 (t, *J* = 6.9 Hz, 2H), 3.33 (t, *J* = 6.9 Hz, 2H), 2.89 (dt, *J*= 12.1, 5.9 Hz, 1H), 2.77-2.67 (m, 2H), 2.42 (ddd, *J* = 19.7, 11.2, 8.8 Hz, 1H), 2.29 (dt, *J* = 12.8, 7.6 Hz, 1H), 2.22-2.13 (m, 1H), 1.82 (dt, *J* = 13.0, 4.8 Hz, 1H), 1.68-1.32 (m, 7H), 1.23 (q, *J* = 5.3 Hz, 1H), 1.13 (q, *J* = 11.4 Hz, 1H), 1.02 (t, *J* = 7.3 Hz, 3H), 0.98 (t, *J* = 7.4 Hz, 3H). ^{13}C-NMR (100 MHz, $CDCl_3$) δ_c: 172.7, 170.7, 168.3, 138.6, 134.5, 73.5, 41.0, 39.1, 37.7, 37.4, 33.4, 29.6, 29.1, 27.8, 26.9, 21.5, 20.8, 13.2, 11.2, 3.20; HRMS (ESI, positive) *m/z* $[M+Na]^+$ Calcd.for $C_{20}H_{29}IN_2O_4Na$ 511.1069, Found 511.1028.

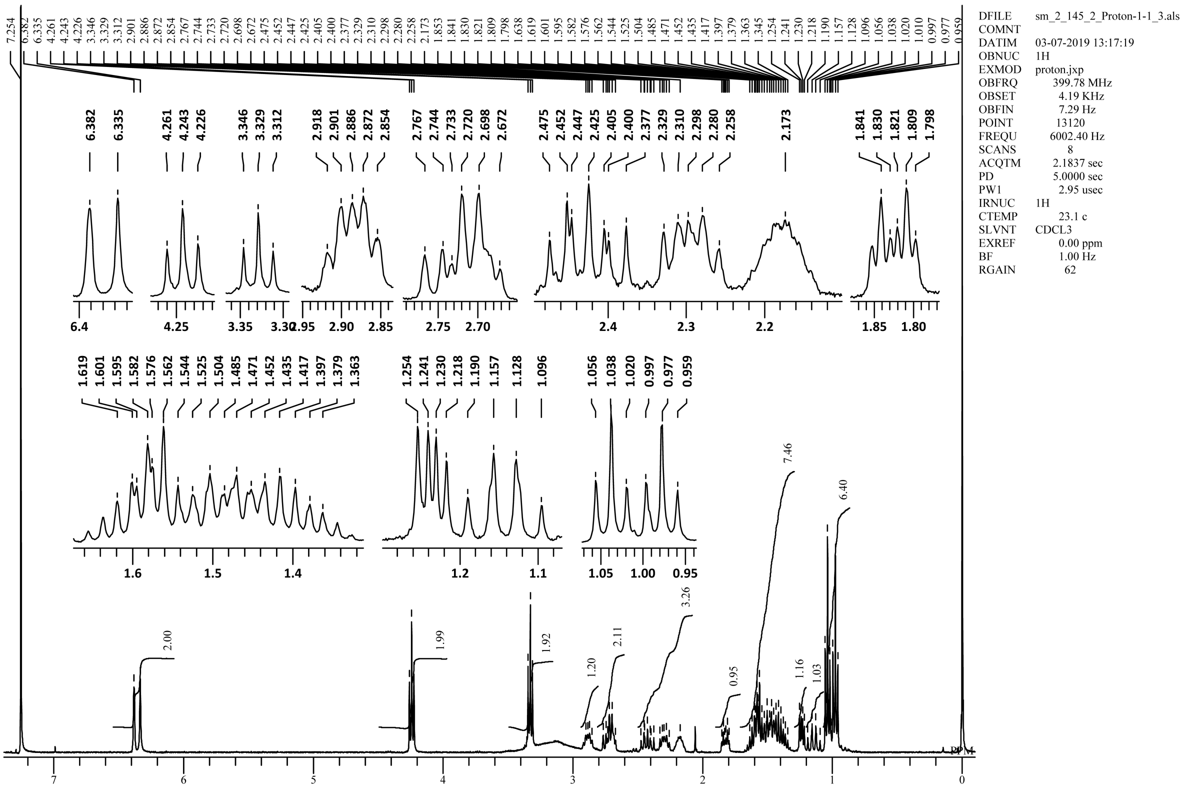

^{1}H NMR spectrum of compound **7**.

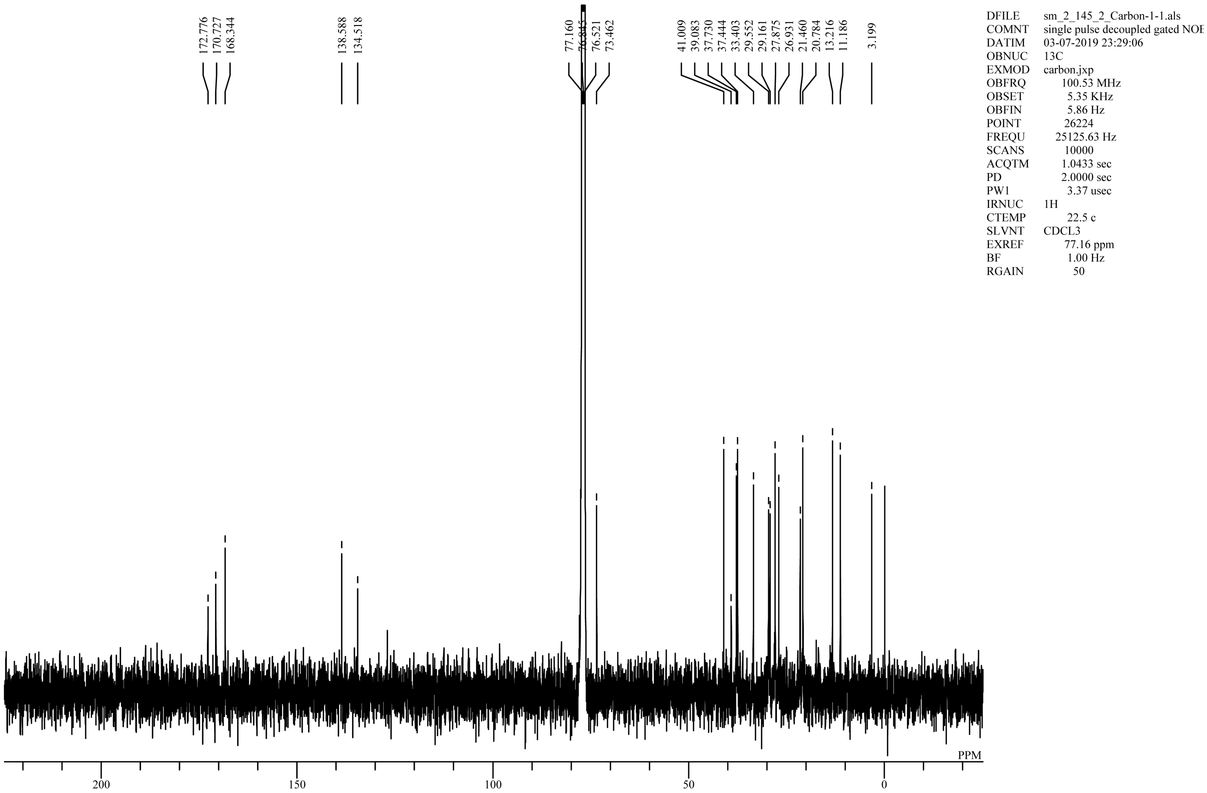

^{13}C NMR spectrum of compound **7**.

Synthesis and characterization of S2:

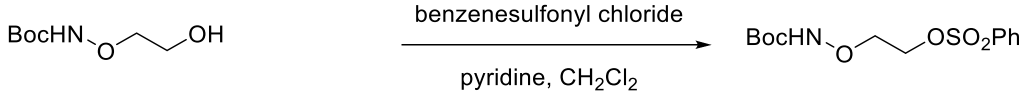

1,1-Dimethylethyl *N*-(2-hydroxyethoxy)carbamate **S2**

To a solution of 1,1-dimethylethyl *N*-(2-hydroxyethoxy)carbamate (214 mg, 1.21 mmol) in dehydrated pyridine (1.2 mL) and CH_2Cl_2 (1.2 mL) was added benzenesulfonyl chloride (256 mg, 1.45 mmol) at 0 °C. After being stirred at r.t. for 1 h, the reaction mixture was quenched with brine, and the mixture was extracted with EtOAc. The combined organic layer was washed with1 M aqueous HCl and then brine, dried over Na_2SO_4, and then concentrated *in vacuo.* The residue was purified by flash chromatography (*n*-hexane/EtOAc = 19/1 to 1/1) to afford **S2** (210 mg) as a colorless oil: ^{1}H NMR (400 MHz, $CDCl_3$) δ_H: 7.92 (dd, *J* = 8.5, 1.5 Hz, 2H), 7.67 (tt, *J* = 7.4, 1.5 Hz, 1H), 7.56 (t, *J* = 7.4, 2H), 7.36 (s, 1H), 4.29 (t, *J* = 4.5 Hz, 2H), 4.04 (t, *J* = 4.5 Hz, 2H), 1.46 (s, 9H); ^{13}C NMR (100 MHz, $CDCl_3$) δ_C: 156.7, 135.7, 133.8, 129.2 (2C), 127.7 (2C), 81.9, 73.2, 67.2, 28.0 (3C); HRMS (ESI, positive) *m*/*z* [M+Na]$^+$ Calcd for $C_{13}H_{19}NO_6SNa$ 340.0825, Found 340.0823.

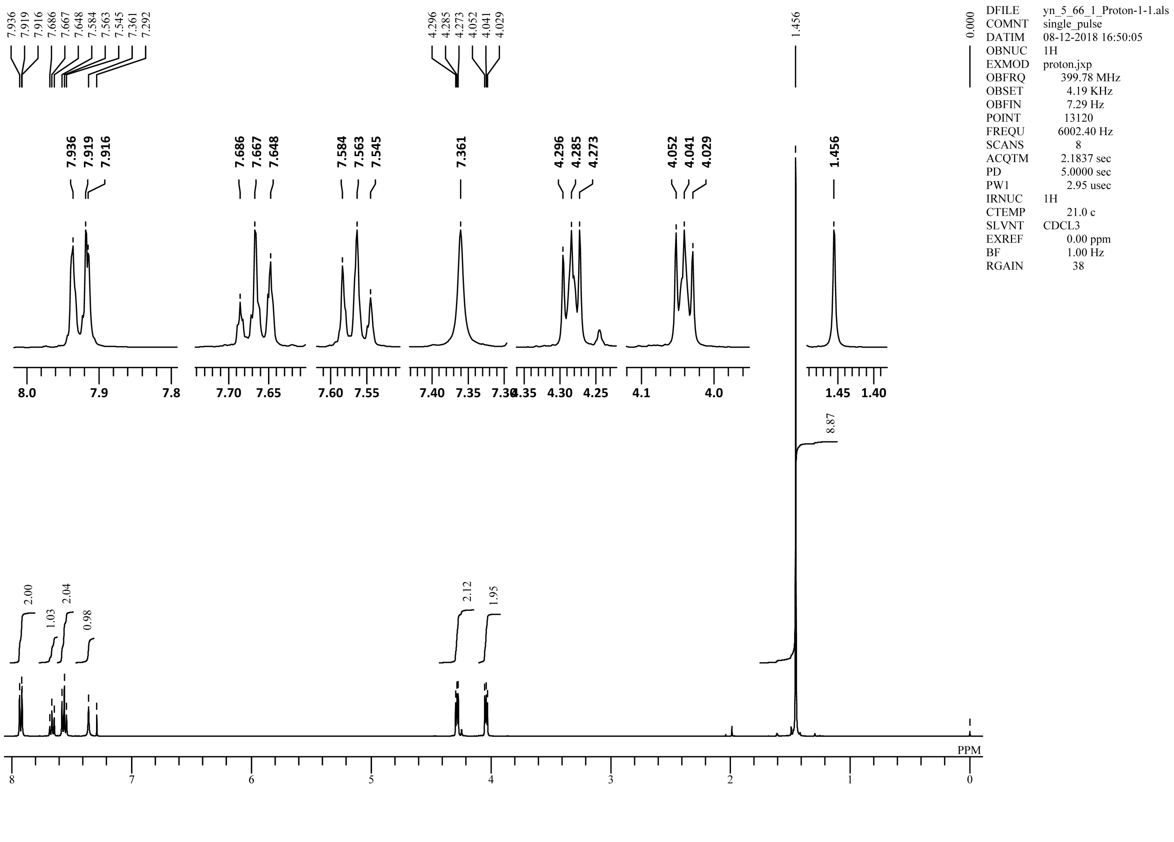

^{1}H NMR spectrum of compound **S2**.

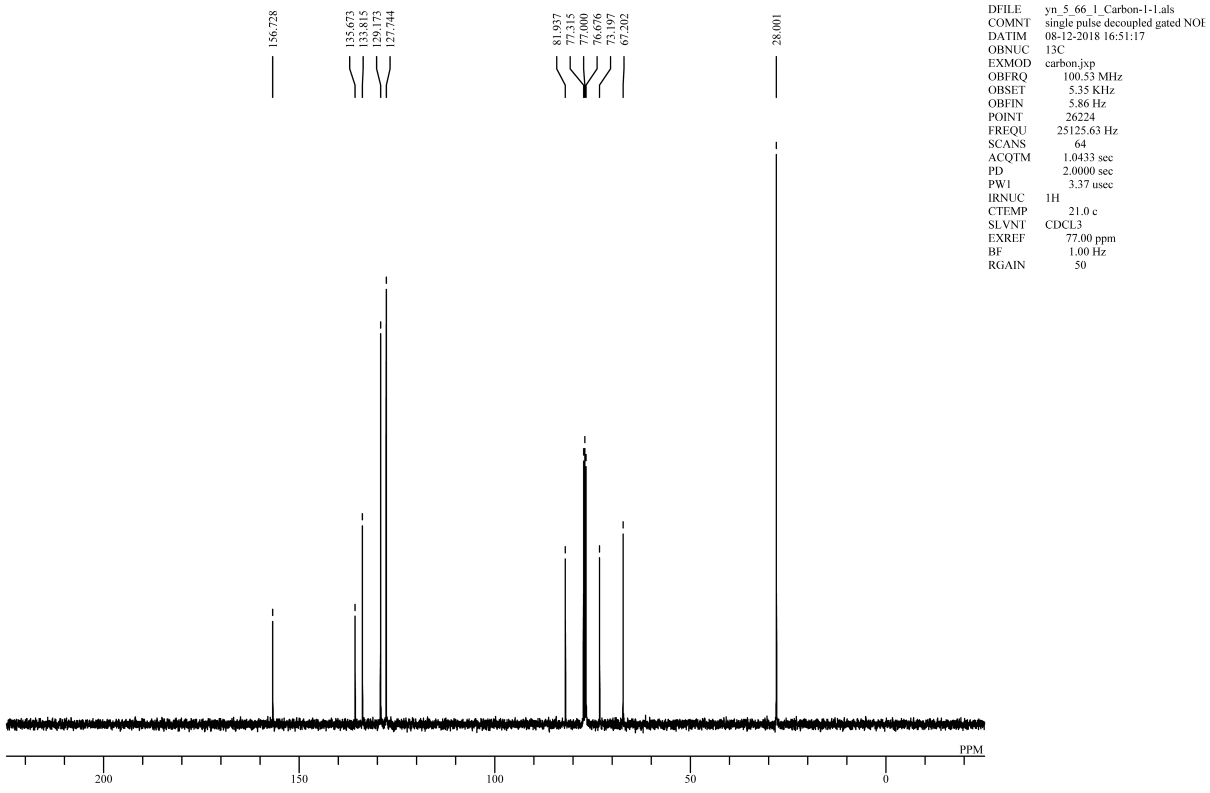

^{13}C NMR spectrum of compound **S2**.

Synthesis of S3:

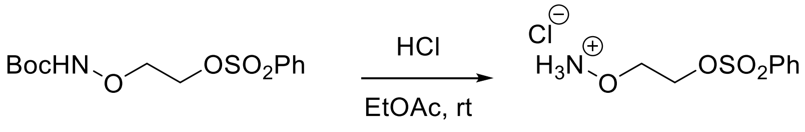

S2 **S3**

Compound **S2** (134 mg, 421 μmol) was treated with 4 M HCl in EtOAc (3 mL) at r.t. for 45 min. The mixture was concentrated *in vacuo* to afford crude **S3** (141 mg), and was used for next step without further purification.

Synthesis and characterization of 8:

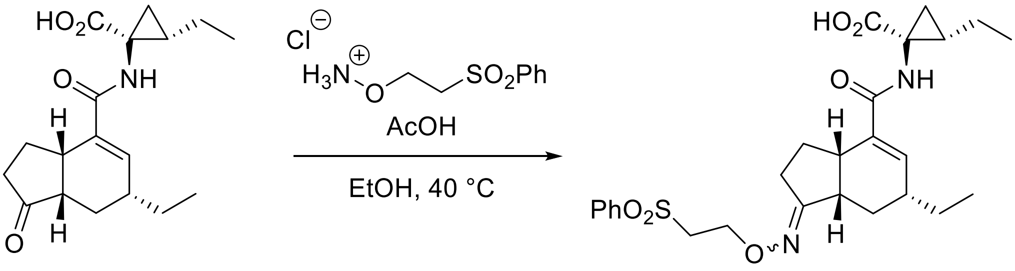

COR (2) **8**

COR (**2**) (0.96 mg, 3.0 μmol) and excess amount of crude **S3** (8.9 mg, ca. 35 μmol) were added to a solution of 0.05 M AcOH in EtOH (0.4 mL). The mixture was stirred at 40 °C for 1.5 h. Then, the mixture was concentrated *in vacuo* and the residue was purified by HPLC equipped with COSMOSIL Cholester column (ϕ20 × 250 mm, Nacalai Tesque co., ltd.) with 0.05% TFA solution (CH_3CN / H_2O = 50 / 50) at flow rate of 10.0 mL/min, the ratio of oxime isomers was about 2:1, Rt = 30 min as major isomer, Rt = 26 min as minor isomer) to afford **8** (0.8 mg, 51% for major isomer; 0.1 mg, 6% for minor isomer) as a colorless oil. The major isomer was used for the assays. major isomer: ^{1}H-NMR (400 MHz, $CDCl_3$) δ_H :7.91 (d, *J*= 7.89, 2H), 7.65 (t, *J* = 7.89, 1H), 7.54 (t, *J* = 7.89, 2H), 6.39 (s, 1H), 6.32 (s, 1H), 4.26 (t, *J*= 6.9 Hz, 2H), 4.20 (t, *J* = 6.9 Hz, 2H), 2.82 (dt, *J* = 12.1, 5.9 Hz, 1H), 2.67-2.55 (m, 2H), 2.36-2.22 (m, 2H), 2.20-2.10 (brs, 1H), 1.76 (dt, *J* = 13.0, 4.8 Hz, 1H), 1.67-1.33 (m, 7H), 1.20 (q, *J* = 5.3 Hz, 1H), 1.08 (q, *J* = 11.4 Hz, 1H), 1.05 (t, *J* = 7.3 Hz, 3H), 0.98 (t, *J* = 7.4 Hz, 3H). ^{13}C-NMR (100 MHz, $CDCl_3$) δ_C: 174.8, 170.4, 168.9, 138.2, 135.9, 134.9, 133.7, 129.2 (2C), 127.9 (2C), 70.6, 68.7, 41.0, 39.3, 37.8, 37.5, 33.6, 29.7, 29.1, 28.0, 26.9, 22.3, 20.7, 13.4, 11.4. HRMS (ESI, positive) *m/z* $[M+Na]^+$ Calcd.for $C_{26}H_{34}N_2O_7SNa$ 541.1979, Found 541.1979.

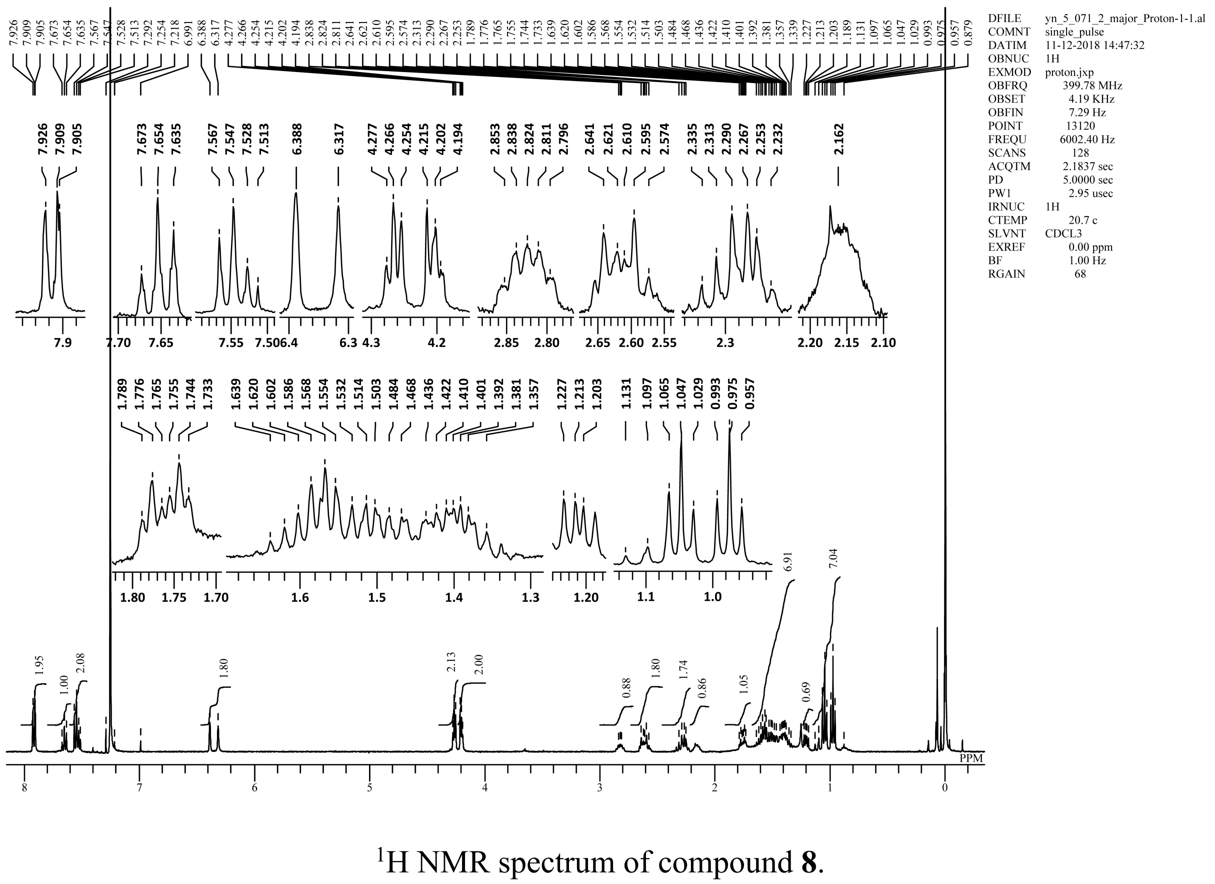

^{1}H NMR spectrum of compound **8**.

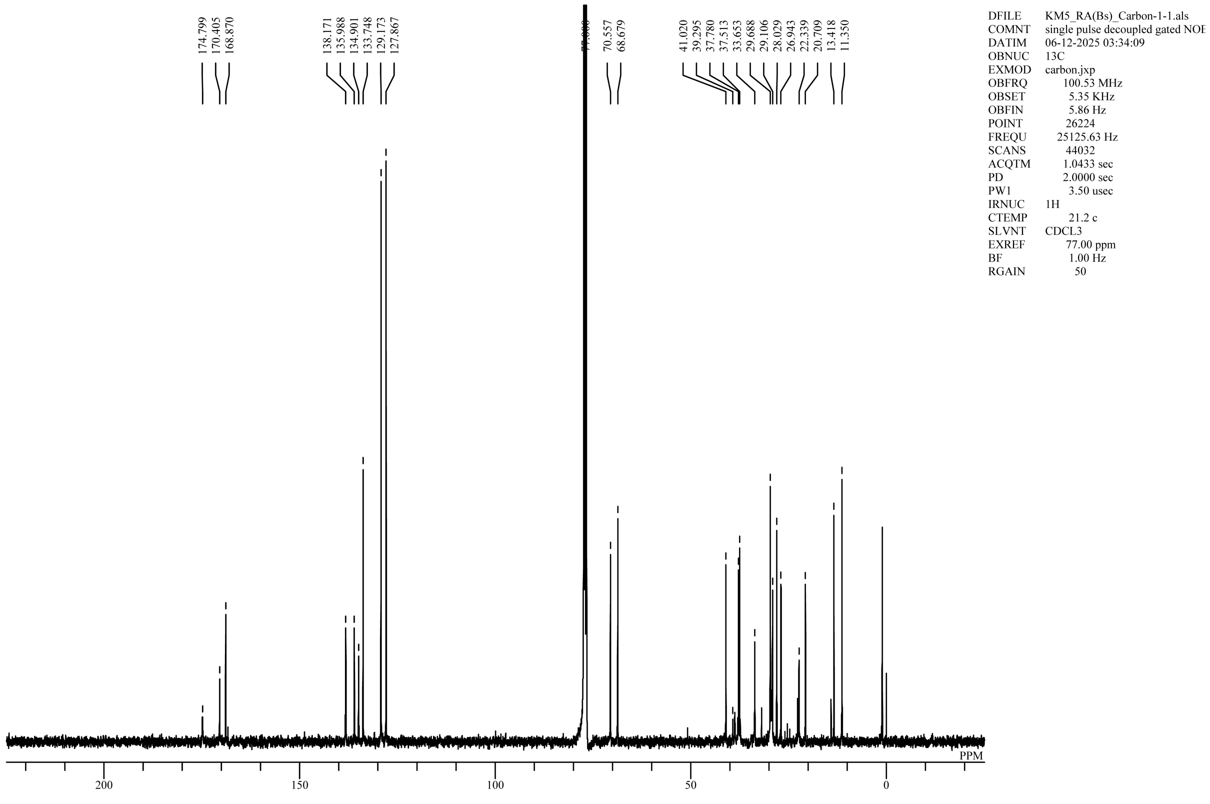

^{13}C NMR spectrum of compound **8**.

Synthesis and characterization of 9:

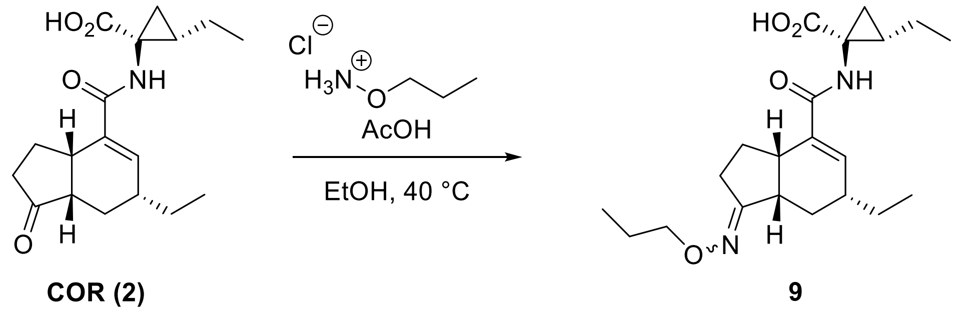

COR (**2**) (2.4 mg, 7.5 μmol) and 1-(Aminooxy)propane hydrochloride (20 mg, 0.27 mmol) were added to a solution of 0.05 M AcOH in EtOH (0.3 mL). The mixture was stirred at 40 °C for 30 min. Then, the mixture was concentrated *in vacuo* and residue was purified by HPLC equipped with COSMOSIL Cholester column (ϕ20 × 250 mm, Nacalai Tesque co., ltd.) with 0.05% TFA solution (CH_3CN / H_2O = 48 / 52) at flow rate of 10.0 mL/min, the ratio of oxime isomers was about 2:1, Rt = 36 min as major isomer, Rt = 34 min as minor isomer) to afford **9** (1.5 mg, 53% for major isomer; 0.8 mg, 28% for minor isomer) as a colorless oil. The major isomer was used for the assays. major isomer: ^{1}H-NMR (400 MHz, $CDCl_3$) δ_H: 6.39 (s, 1H),

6.32 (s, 1H), 4.00 (t, *J* = 6.9 Hz, 2H), 2.86 (dt, *J* = 12.1, 5.9 Hz, 1H), 2.76-2.64 (m, 2H), 2.44-2.34 (m, 1H), 2.34-2.24 (m, 1H), 2.22-2.12 (brs, 1H), 1.85 (dt, *J* = 13.0, 4.8 Hz, 1H), 1.65 (sext, *J* = 6.8 Hz, 2H), 1.60-1.32 (m, 7H), 1.26-1.90 (m, 1H) 1.2 (q, *J* = 14.45 Hz, 1H), 1.03 (t, *J* = 6.8 Hz, 3H), 0.97 (t, *J* = 7.3 Hz, 3H), 0.93 (t, *J* = 7.4 Hz, 3H). ^{13}C-NMR (100 MHz, CD_3OD) δ_c: 172.0, 169.3, 168.7, 138.5, 136.3, 76.1, 42.4, 39.1, 38.9, 33.1, 30.9, 30.1, 29.2, 27.6, 26.7, 23.4, 23.2, 21.7, 13.8, 11.6, 10.7; HRMS (ESI, positive) *m*/ $[M+Na]^+$ Calcd.for $C_{21}H_{32}N_2O_4Na$ m/z 399.2255, Found 399.2254.

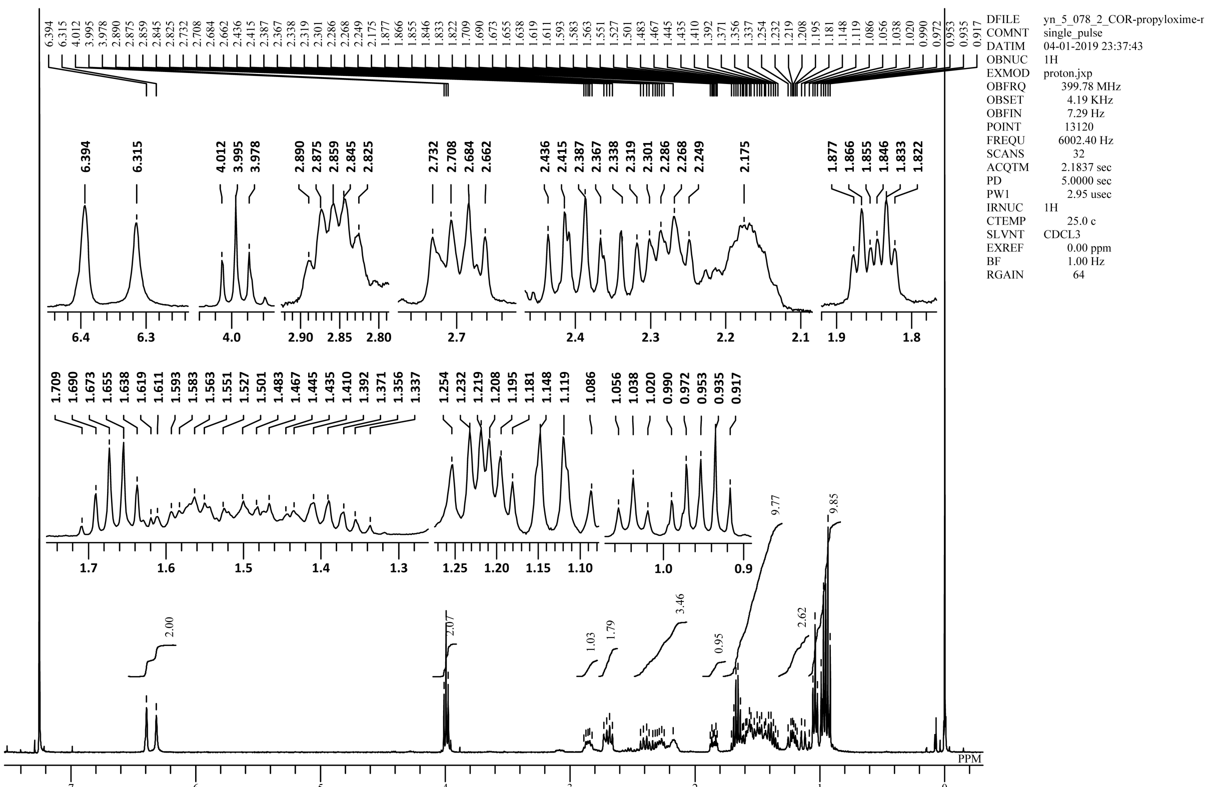

^{1}H NMR spectrum of compound **9**.

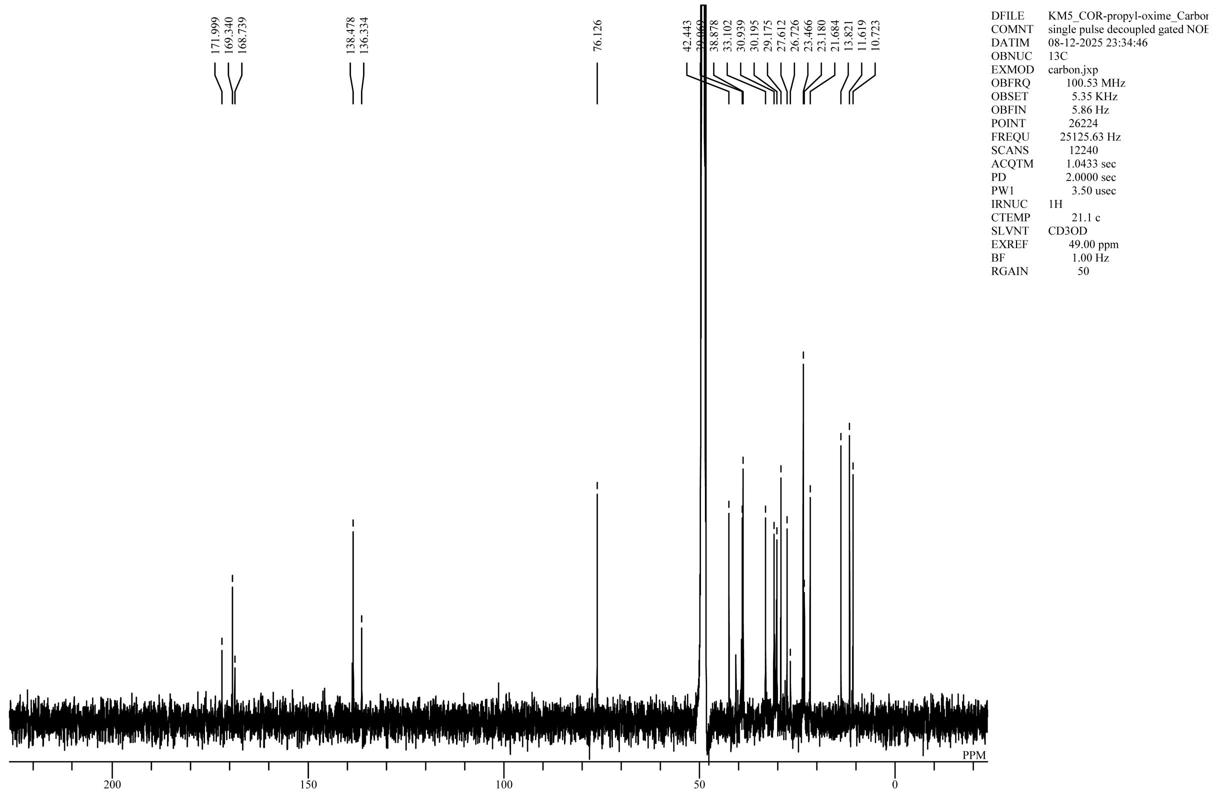

^{13}C NMR spectrum of compound **9**.

Synthesis of JAZ peptides: Fluorescein-conjugated wild-type JAZ peptides (Fl-JAZ1-13P) has been already reported.[S4] Cys-mutated JAZ peptides (Fl-^{I203C}JAZ1P and Fl-^{I205C}JAZ2P) were newly prepared by microwave-assisted solid phase synthesis with NovaSyn® TGA regin (90 μm) using Initiator+ Alstra (Biotage Ltd., US) as previously described.[S4] The purity of these peptides was confirmed by HPLC analysis, and these were characterized by MALDI-TOF/MS as follows (**Figure S16**);

Fl-^{I203C}JAZ1P: m/z $[M+H]^+$ calcd for 3585.82, found 3585.81

Fl-^{I205C}JAZ2P: m/z $[M+H]^+$ calcd for 3599.83, found 3599.84

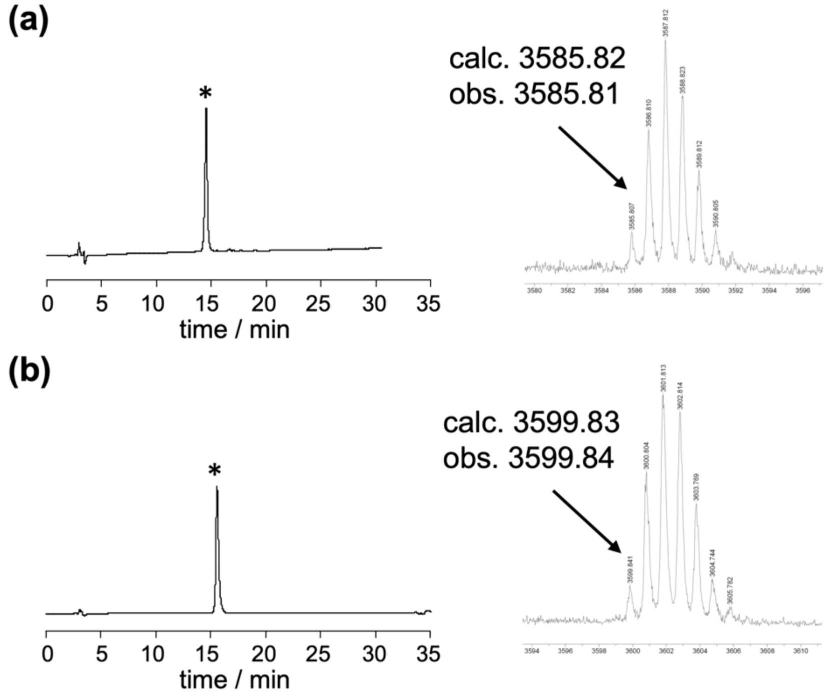

Figure S16. HPLC charts (left) and MALDI-TOF MS spectra (right) of the purified peptides synthesized in this study; **(a)** Fl-^{I203C}JAZ1P, Fl-^{I205C}JAZ2P.

Preparation of plant materials (*P35S::^{I203C}JAZ1-GFP*, *P35S::^{I205C}JAZ2-GFP*, *P35S::^{I140C}JAZ12-GFP*): The vector *pGWB5-JAZ2* was kindly provided from Prof. Dr. R. Solano and Dr. Andrea Chini (CNB-CSIC, Madrid, Spain). To create *P_{35S}:: ^{I205C}JAZ2-GFP* transgenic Arabidopsis lines, at first, *P_{35S}::JAZ2-GFP* gene was amplified by PCR (primers 1&2 shown in Table S1) from *pGWB5-JAZ2*, and the PCR product was transferred into the Sal I-Xho I site of pET-32a (Invitrogen) to generate *pET-N32a-P_{35S}::JAZ2-GFP*. In addition, short gene segment containing some restriction enzyme sites (EcoR I, Sal I, and Xho I; created with primers 3 and 4) was inserted into Hind III-Sac I site of *pGWB5-JAZ2* to generate *pGWB_adaptor*. Then, point mutation was introduced by inverse PCR with primers 5 and 6, generating *pET-N32a-P_{35S}::^{I205C}JAZ2-GFP*. Finally, *P_{35S}-^{I205C}JAZ2-GFP* was transferred into the Sal I-Xho I site of *pGWB5_adaptor* to generate *pGWB5-P35S::^{I205C}JAZ2-GFP*.

To obtain transgenic *Arabidopsis* lines expressing *P_{35S}::JAZ1-GFP*, *P_{35S}::JAZ12-GFP*, *P_{35S}::^{I203C}JAZ1-GFP*, and *P_{35S}::^{I140C}JAZ12-GFP*, at first, each JAZ gene from *pDESTH1-AtJAZ1* and *pDESTH1-AtJAZ12* (also kindly gifted from Prof. R. Solano and Dr. Andrea Chini), and *pET-N32a-P_{35S}::JAZ2-GFP* vector containing the 35S promoter and GFP gene was amplified by PCR respectively (Primers 7–10 shown in Table S1). Point mutation was introduced by inverse PCR (primers 11-14), from *pET-N32a-35S::JAZ-GFP* generating *pET-N32a-35S::cysJAZ-GFP*. These were ligated using In-Fusion Cloning Kit (Clontech, Takara

Bio USA, Inc. CA, USA) to obtain *pET-N32a-P_{35S}::JAZ-GFP*. The *P_{35S}::JAZ-GFP* was then amplified from *pET-N32a-35S::JAZ-GFP* (primers 15&16). From this, *P_{35S}::JAZ-GFP* amplified by PCR (primers 17&18) and introduced into the Sal I–Xho I site (for JAZ1) or Hind III–Sac I site (for JAZ12) of *pGWB5_adapter* by restriction enzymes to obtain *pGWB5-35S::JAZ-GFP*.

Agrobacterium (GV3101) was transfected with *pGWB5-35S::JAZ-GFP* or *pGWB5-35S::^{cys}JAZ-GFP* plasmid by electroporation (200 Ω, 25 μF, 2500 V). After 2h incubation in SOC medium at 28 ºC, the cells were incubated at 28 °C for 2 days on LB agar with 50 μg/mL kanamycin. Single colonies were picked up and incubated in 3 mL of LB liquid medium with 50 μg/mL kanamycin at 30 °C for 24 h (150 rpm). The agrobacterium-culture was then placed in 200 mL of LB liquid medium with 50 μg/mL kanamycin to reach OD600 = 0.001 and incubated at 30 °C until OD600 reached to 1.0–1.5 (150 rpm), and then the cell suspension was collected with centrifugation (4000 g, 15 min at r.t.). Obtained agrobacterium cell pellets was suspended in 3 mL of Dip buffer (10 g sucrose, 100 mg MES in 200 mL distilled water), and then diluted with the same Dip buffer to adjust the OD600 value to around 0.8 (~150 mL). Then 50 μL of SILWET L-77 (Momentive Performance Materials, Inc., New York, US) and 2 μL of 1 mg/mL 6-Benzyladenine (BAP, Fujifilm Wako Pure Chemical Industries Co., Ltd., Tokyo, Japan) was added to the agrobacterium cell suspension (= Dip solution).

Flowers and pods of 6–8 week old Col-0 plants were cut and then buds were dipped in the above obtained Dip solution under reduced pressure for 10 min. Buds were covered with plastic wrap and plants were lay down under a 24 h light (2080 lux, cool-white fluorescent light) at 22°C in a Biotron LH300 growth chamber (Nippon Medical & Chemical Instruments Co., Ltd., Osaka, Japan). After 24 hours, the buds were allowed to stand and grown until seeds were ready. (As for ^{wt}JAZ1, 2, 12 and ^{cys}JAZ1, 2 were transformed by INPLANTA INNOVATIONS Inc. Japan). 1st generation of the transgenic line (T1) seeds were obtained from the grown plants by selection on 1/2 MS medium with 20 μg/mL hygromycin. T2 seeds were grown from those for which nuclear localization of JAZ-GFP was confirmed by fluorescence imaging (Carl Zeiss Co., Ltd., Germany). The resulting T2 lines were selected on 1/2 MS medium 20 μg/mL hygromycin. JAZ-GFP expression levels were checked by fluorescence imaging and western blotting analyses, and T3 was picked up from lines with high expression levels. In this work, we used T2 or T3 lines that were confirmed to show the sufficient expression levels.

Plant growth condition: *A. thaliana* ecotype Col-0 and all mutant seeds were surface-sterilized in 5% sodium hypochlorite with 0.3% Tween-20 and vernalized for 2-3 days at 4 ºC. All

seedlings were grown under a 16 h light (2100 lux, cool-white fluorescent light)/8 h dark cycle at 22 ºC in a Biotron NC-220 growth chamber (Nippon Medical & Chemical Instruments Co., Ltd., Osaka, Japan). WT and all mutant seedlings were vertically grown in 1/2 Murashige and Skoog (MS) solid media for 4-6 days.

Supplementary References

S1) Kato, N. *et al*. A scalable synthesis of (+)-coronafacic acid. *Chirality* **32**, 423–430 (2020).

S2) Nara, S., Toshima, H. & Ichihara, A. Phytotoxins Isolated from Pseudomonas Syringae. *Pathovars.* **53**, 9509–9524 (1997).

S3) Bauer, L. & Suresh, K. S. S-[ω-(Aminoöxy)alkyl]isothiuronium Salts, ω,ω′-Bis(aminoöxy)alkanes and Related Compounds. *J. Org. Chem.* **28**, 1604–1608 (1963).

S4) Takaoka, Y. et al. A comprehensive in vitro fluorescence anisotropy assay system for screening ligands of the jasmonate COI1-JAZ co-receptor in plants. *J. Biol. Chem.* **294**, 5074-5081 (2019).
